# *γ*‐Valerolactone‐Based Anion‐Dominated Loose Solvation Electrolyte Enables Stable Lithium Metal Batteries from −60°C to 100°C

**DOI:** 10.1002/advs.202523560

**Published:** 2026-02-06

**Authors:** Lei Zhang, Tianle Zheng, Qing Ming, Keyu Zheng, Jin Zhu, Yiyao Xiao, Said Amzil, Mengqi Wu, Shengyao Luo, Meilan Peng, Yinghui Li, Xiuxia Zuo, Peter Müller‐Buschbaum, Ya‐Jun Cheng, Yonggao Xia

**Affiliations:** ^1^ School of Materials Science and Chemical Engineering Ningbo University Ningbo Zhejiang P. R. China; ^2^ Ningbo Institute of Materials Technology and Engineering Chinese Academy of Sciences Ningbo Zhejiang P. R. China; ^3^ TUM School of Natural Sciences Department of Physics Chair For Functional Materials Technical University of Munich Garching Germany; ^4^ Jiangsu Cnano Technology Co., Ltd. Zhenjiang Jiangsu P. R. China; ^5^ College of New Energy Ningbo University of Technology Ningbo P. R. China; ^6^ College of Renewable Energy Hohai University Changzhou Jiangsu P. R. China; ^7^ Center of Materials Science and Optoelectronics Engineering University of Chinese Academy of Sciences Beijing P. R. China

**Keywords:** anion‐dominated solvation structure, extreme temperature electrolytes, fast desolvation kinetics, high‐energy‐density lithium‐metal batteries

## Abstract

Integrating high‐nickel cathodes with lithium metal anodes enables ultrahigh‐energy‐density batteries but remains challenged by electrolyte instability under extreme temperatures. Here, we design an anion‐dominated loose solvation structure, where fluorine‐rich weak solvents occupy coordination sites. *γ*‐Valerolactone (GVL) serves as the primary solvent, assisted by two weakly coordinating co‐solvents: difluoroethylene carbonate (DFEC) to modulate solvation and ethyl trifluoroacetate (ETFA) to reduce viscosity and freezing point. This balanced solvation environment enhances ionic transport, interfacial stability, and desolvation kinetics. Consequently, Li||NCM811 cells deliver stable cycling at 100°C, exceeding 90 cycles, negligible capacity loss at −40°C, and 90.4 mAh g^−1^ at −60°C. Full cells (N/P ≈ 1.8) retain 90.3% capacity after 130 cycles. This work offers a viable solvation design for high‐voltage lithium metal batteries operating across extreme temperatures.

## Graphical abstract

The precise regulation of GVL's solvation structure by two weak solvents with distinct functions promotes the formation of an anion‐dominated loose solvation structure, enabling the stable generation of EEI to protect the structural integrity of cathode materials and promote the uniform deposition of lithium.

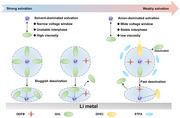

## 1 Introduction

The pursuit of high‐energy‐density rechargeable batteries with robust safety and wide‐temperature tolerance has stimulated extensive research on lithium metal batteries (LMBs) coupled with high‐nickel cathodes such as LiNi_0.8_Co_0.1_Mn_0.1_O_2_ (NCM811) [1]. Lithium metal anodes offer an ultrahigh theoretical capacity (3860 mAh g^−^^1^) and the lowest redox potential (−3.04 V vs. SHE), but their practical application is limited by electrolyte instability, which causes uncontrolled interfacial reactions, dendrite growth, and cathode degradation under extreme conditions [2, 3, 4, 5] These challenges are fundamentally rooted in the solvation structure of the electrolyte, which governs ion transport, interphase composition, and redox stability at both electrodes [6, 7, 8].

Conventional carbonate‐based electrolytes exhibit strong Li^+^‐solvent coordination and a narrow liquid temperature range, resulting in sluggish desolvation kinetics at low/elevated temperatures and severe oxidative decomposition at high voltages [9, 10]. Recent electrolyte engineering strategies, such as high‐concentration or localized high‐concentration electrolytes, have partially mitigated these problems by promoting anion participation in the primary Li^+^ solvation shell [11, 12, 13]. However, these systems often suffer from high viscosity, high cost, and poor wettability, leading to limited performance under wide‐temperature operation [14]. Therefore, constructing an anion‐dominated loose solvation structure where weakly coordinating solvents occupy outer coordination shells while anions approach Li^+^ more closely and enhance its participation in the solvation sheath represents an effective route to balance desolvation kinetics, interfacial stability, and ion mobility.

In this context, *γ*‐valerolactone (GVL) emerges as a promising primary solvent candidate. GVL is a cyclic carboxylate with a wide liquid range (−31°C to 207°C), high dielectric constant, excellent lithium salt solubility, and intrinsic environmental benignity [15, 16]. Nevertheless, its strong coordination with Li^+^ and narrow electrochemical window have hindered its use in high‐voltage LMBs [17, 18]. To overcome these intrinsic limitations, a rational co‐solvent design is required to loosen GVL's solvation ability and tune Li^+^‐anion interactions.

**SCHEME 1:**
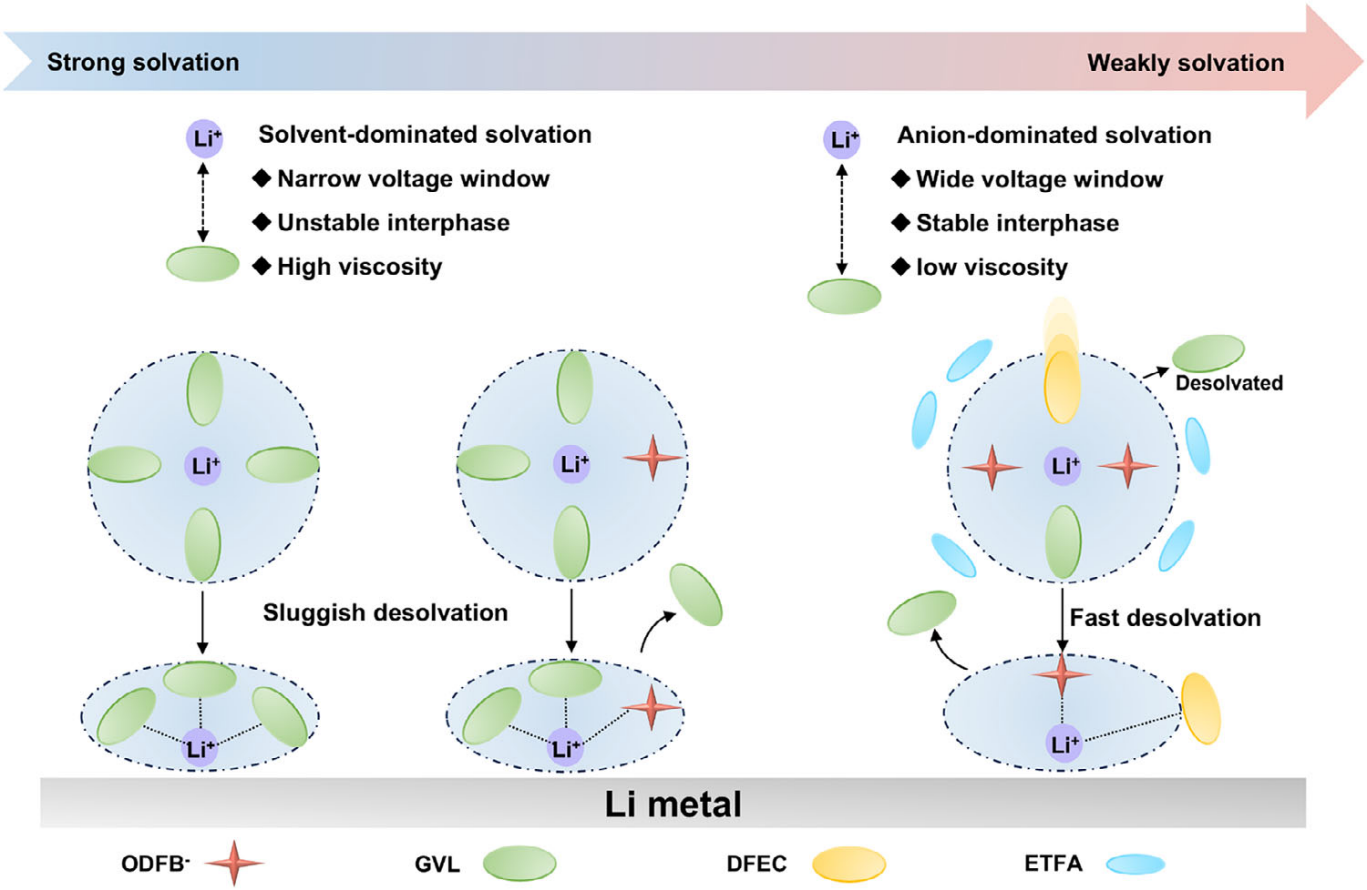
Explanation of the different solvation structures: solvent‐dominated and anion‐dominated.

Among emerging lithium salts, lithium oxalyldifluoroborate (LiODFB) exhibits significant promise as a single‐salt candidate owing to its balanced solubility, excellent current collector compatibility, and effective film‐forming capabilities [19, 20, 21]. Moreover, previous studies have shown that its solvating ability is much weaker than that of several other common lithium salts, which increases the operability for us to subsequently regulate the solvation environment of GVL around Li^+^ [22]. Here we introduce two fluorine‐rich weakly coordinating co‐solvents—difluoroethylene carbonate (DFEC) and ethyl trifluoroacetate (ETFA)—to regulate the solvation landscape of a GVL‐based electrolyte. DFEC possesses moderate donor strength and a five‐membered cyclic structure analogous to GVL, enabling it to partially compete for Li^+^ coordination and perturb the primary solvation sheath. In contrast, ETFA exhibits extremely weak coordination ability but an ultralow freezing point (−78°C) and low viscosity, serving to expand the liquidus range, facilitate ion mobility, and act as a pseudo‐diluent for LiODFB (Scheme 1). Based on this, DFEC mitigates the strong binding between Li^+^ and GVL and partially displaces GVL from the solvation sheath, while ETFA significantly reduces the system viscosity and pushes LiODFB in the system close to its solubility limit—facilitating the entry of a large number of ODFB^−^ anions into the solvation sheath. Ultimately, under the synergistic effect of DFEC and ETFA, an anion‐dominated loose solvation structure is formed.

This tailored solvation configuration achieves a delicate balance among viscosity, ionic conductivity, interfacial stability, and desolvation kinetics. As a result, the optimized electrolyte enables high‐voltage Li||NCM811 cells to cycle stably across an ultra‐wide temperature range (−60°C to 100°C) while maintaining high capacity retention and interphase robustness. This work establishes a molecular‐level solvation modulation strategy based on weakly solvated fluorinated solvents, offering a new paradigm for designing extreme‐temperature lithium metal batteries.

## 2 Result and Discussion

### 2.1 Physicochemical Properties and Solvation Structure of Electrolytes

The optimized 0.8 M LiODFB in GVL/ETFA/DFEC (3:5:2 v/v/v) formulation, denoted as GED electrolyte, exhibits ideal dissolution (Figure S1). Control electrolytes include: 0.8 M LiODFB in GVL/DFEC (8:2 v/v, denoted as GD), 0.8 M LiODFB in GVL/ETFA (5:5 v/v, denoted as GE), commercial electrolyte (1 M LiPF_6_ in EC/DMC = 3:7 v/v, denoted as CE), and 0.8 M LiODFB in GVL (denoted as 0.8G). This work first investigates fundamental properties of GED electrolyte to validate its feasibility under extreme temperatures. Differential scanning calorimetry (DSC) reveals no distinct exothermic peaks in GED down to −100°C (Figure S3), indicating liquid state maintenance, while the commercial electrolyte exhibits solidification behavior near −22°C. Additionally, conductivity and viscosity measurements at 25°C (Table S1) reveal that the GED electrolyte maintains low viscosity while delivering suitable conductivity.

**FIGURE 1:**
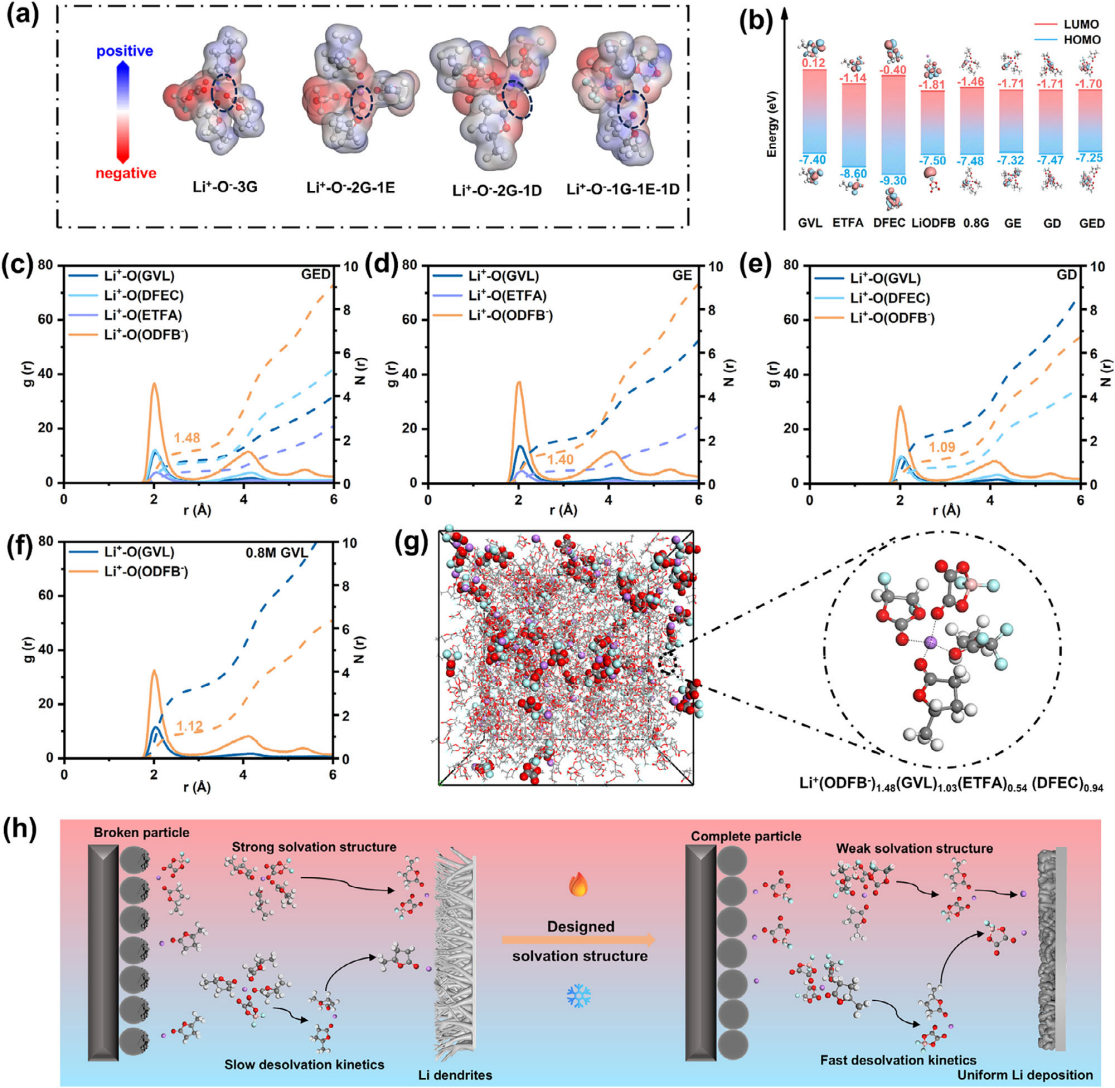
(a) Electrostatic potential mappings of Li^+^‐1ODFB^−^‐3GVL, Li^+^‐1ODFB^−^‐2GVL‐1ETFA, Li^+^‐1ODFB^−^‐2GVL‐1DFEC, and Li^+^‐1ODFB^−^‐1GVL‐1ETFA‐DFEC based on the electron density. (b) HOMO and LUMO energy levels of the solvents, lithium salt, and electrolyte. (c–f) Radial distribution function and coordinated number of different electrolytes at 25°C. (g) MD simulation box and representative solvation structures extracted from MD simulation boxes of GED electrolyte at 25°C. (h) Schematic diagram of electrolyte solvation structure optimizing interface and altering desolvation process.

Electrostatic potential (ESP) calculations (Figure S5) identify a concentrated negative charge on GVL's carbonyl oxygen. Due to fluorine's strong electron‐withdrawing effect, DFEC (ESP_min_ = −30.3714 kcal·mol^−1^) and ETFA (ESP_min_ = −34.8895 kcal·mol^−1^) exhibit substantially higher ESP_min_ than GVL (ESP_min_ = −47.2515 kcal·mol^−1^), indicating GVL's strongest Li^+^ binding affinity. Binding energy calculations confirm this hierarchy (Figure S6), establishing the solvent desolvation capability order as ETFA > DFEC >> GVL. Additionally, the ESP calculations of solvation structures further verify differences in desolvation kinetics (Figure 1a). Clear observation indicates that in the Li^+^‐O^−^‐1G‐1E‐1D structure, the introduction of the other two solvents significantly reduces the negative charge on GVL's C = O group and disrupts its strong shielding effect on Li^+^. This critically promotes ODFB^−^ participation in the solvation structure.

Density functional theory (DFT) calculations of highest occupied molecular orbital (HOMO) and lowest unoccupied molecular orbital (LUMO) energies for electrolyte components reveal that a narrow electrochemical window of LiODFB causes it to undergo irreversible decomposition reactions at both electrodes, forming inorganic‐rich interfacial films (Figure 1b). Since ETFA and DFEC have higher HOMO energy levels compared to GVL, incorporating these solvents into the electrolyte to modulate Li^+^ solvation can effectively suppress GVL over‐oxidation at the cathode, as confirmed by the linear sweep voltammetry (LSV) tests (Figure S7). Additionally, the solvation structure of GED exhibits lower HOMO and LUMO energy levels, which facilitates preferential anion decomposition to form a stable Electrode‐Electrolyte Interphase (EEI) while simultaneously suppressing continuous solvent decomposition.

Molecular dynamics (MD) simulations of the electrolytes reveal distinct solvation structures via radial distribution functions (RDFs) (Figure 1c–f). The evolution of the Li^+^ coordination numbers (right *y*‐axis) with solvents and anions indicates that in pure GVL dissolving LiODFB, Li^+^ primary solvation shells are predominantly composed of GVL molecules, with minimal ODFB^−^ coordination. Upon introducing ETFA, although ETFA itself shows negligible coordination, it promotes increased ODFB^−^ coordination with Li^+^, raising ODFB^−^ coordination number (CN) from 1.12 to 1.40. However, GVL remains dominant in the primary solvation shell. After the introduction of DFEC molecules, a competitive coordination effect can be observed due to DFEC's strong coordination ability. This effect leads to the exclusion of more GVL molecules from the primary solvation shell and promotes the involvement of some ETFA in coordination, ultimately forming a solvation structure dominated by ODFB^−^ ions in the GED electrolyte. Moreover, the CN of GVL molecules with Li^+^ changes from 3.25 in the initial pure solvent to 1.03 (Figure 1g; Figure S9), which greatly reduces the excessive oxidation of GVL. High‐temperature MD simulations (T = 100°C) confirm exceptional stability with no observable changes in GED's solvation structure (Figures S10 and S11). The effect of the optimized solvation structure on the cathode and anode interfaces, as well as the improvement in desolvation kinetics, is shown in Figure 1h.

**FIGURE 2:**
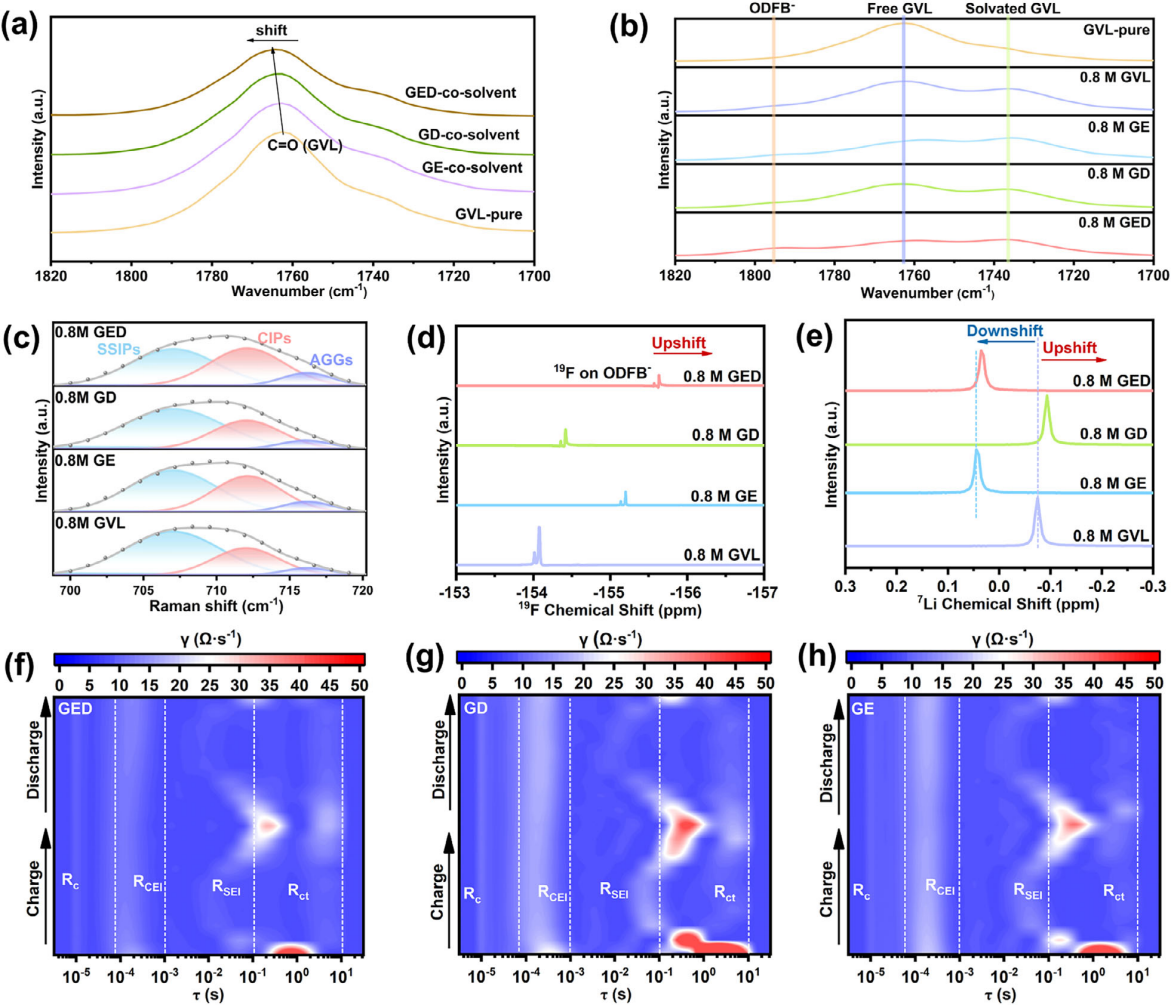
(a) IR spectra of GVL, GVL‐ETFA, GVL‐DFEC, and GVL‐ETFA‐DFEC mixed solution in 1700–1800 cm^−1^. (b) IR spectra of various electrolytes in 1700–1820 cm^−1^. (c) Raman spectra of various electrolytes in 700–720 cm^−1^. (d) ^7^Li‐NMR spectra of various electrolytes. (e) ^19^F‐NMR spectra of the ODFB^−^ in various electrolytes. In‐situ DRT analysis of Li||NCM811 Cells with (f) GED electrolyte; (g) GD electrolyte, and (h) GE electrolyte.

Infrared (IR) spectroscopy further confirms intermolecular interactions among the solvents. Figure S12 shows the pure GVL C = O stretching vibration at 1762 cm^−^^1^ and DFEC's characteristic C = O peak near 1850 cm^−^^1^. Figure 2a demonstrates that progressive addition of ETFA and DFEC shifts GVL's C = O peaks to higher wavenumbers in the GED co‐solvent system due to the strong electron‐withdrawing effects of fluorine‐rich groups and orbital overlap interactions. This shift indicates reduced electron density at GVL's carbonyl group. In Figure 2b, the 1762 cm^−^^1^ peak corresponds to free GVL, while LiODFB introduction generates a new peak at 1795 cm^−^^1^ [23] and a dominant 1737 cm^−^^1^ band representing solvated GVL (C = O‐Li^+^ coordination). The intensity of this peak reflects their binding strength. It is found that with the introduction of the two fluorine‐rich weakly solvated solvents, their binding strength gradually decreases, which is consistent with the previous DFT calculation results. In addition, the ^7^Li NMR spectra show that the GED electrolyte eventually exhibits a downfield shift trend (Figure 2d), which indicates an enhancement of the deshielding effect around Li^+^, verifying that the loose solvation structure of GED is beneficial to desolvation. Interestingly, upon the addition of DFEC, the ^7^Li signal shifts slightly upfield, which may be attributed to the compression of the original solvation sheath caused by the interaction between DFEC and GVL [24]. Additionally, the previous RDF calculation results demonstrate that after introducing the DFEC solvent, the radius of the electrolyte's second solvation sheath changed from 4.11 Å in the 0.8G electrolyte to 4.09 Å in the GD electrolyte, which also illustrates this point. Raman spectra (Figure 2c; Figure S14) show that after dissolving the lithium salt in GVL, a characteristic peak of ODFB^−^ appears at around 710 cm^−^^1^. The three different existing forms of ODFB^−^ in the electrolyte are identified as aggregates (AGGs) at approximately 716 cm^−^^1^, contact ion pairs (CIPs) at approximately 712 cm^−^^1^, and solvent‐separated ion pairs (SSIPs) at approximately 707 cm^−^^1^ [25]. We perform fits to the characteristic region of the ODFB^−^ anion in the Raman spectra. The results clearly show that with the introduction of ETFA and DFEC, the proportion of CIPs and AGGs in the electrolyte increases from the original 28.6% and 5.3% to 42.2% and 9.6%, respectively. This finding indicates that more ODFB^−^ anions participate in the solvation structure (Figure S16). This observation aligns with the MD simulation result. ^1^ ^9^F NMR upfield shift of ODFB^−^ further supports this (Figure 2e). Variable‐temperature Raman spectra (Figure S17) show no detectable change in the ODFB^−^ peak region (700–720 cm^−^^1^) from 25°C to 100°C in GED electrolyte, with the solvation structure of GED demonstrating exceptional thermal stability consistent with high‐temperature MD simulations.

To clarify the influence of the GED solvation structure on interphase formation and desolvation kinetics, the dynamic evolution of impedance during cycling is investigated using in‐situ electrochemical impedance spectroscopy (EIS) combined with distribution of relaxation times (DRT) analysis in Li||NCM811 cells (Figure 2f–h; Figure S19). As illustrated, the DRT spectra resolve four distinct processes based on characteristic relaxation time domains: contact resistance (R_c_) around τ ≈ 10^−^^5^ s, cathode‐electrolyte interphase resistance (R_CEI_) within τ ≈ 10^−^^4^–10^−^^3^ s, anode solid‐electrolyte interphase resistance (R_SEI_) spanning τ ≈ 10^−^^3^–10^−^^1^ s, and charge transfer resistance (R_ct_) covering τ ≈ 10^−^^1^–10^1^ s [26, 27]. It is observed that benefiting from the stable interfacial film generated by the anion‐dominated solvation structure of the GED electrolyte, the battery with GED electrolyte exhibits the lowest R_EEI_ (R_SEI_+R_CEI_) during charging and discharging. In fact, this is because the solvation structure of GED is dominated by anions; thus, the components of its interfacial film are derived from the decomposition of more ODFB^−^ anions, which enables the formation of a LiF‐rich interfacial layer with lower impedance and higher stability, ensuring that the battery maintains consistently low impedance throughout the cycling process. In contrast, the high impedance shown by the GD and GE samples may be mainly due to the continuous decomposition of solvents caused by inappropriate solvation structures, which leads to the continuous thickening of the interfacial film. In addition, R_ct_ mainly corresponds to the solvation and desolvation processes of Li^+^. Whether during charging or discharging, the battery with GED electrolyte consistently exhibits lower R_ct_, which validates accelerated desolvation kinetics in this electrolyte system. Thus, it is successfully confirmed that the solvation structure of the GED electrolyte induces the formation of a stable and low‐impedance EEI as well as fast desolvation kinetics.

### 2.2 Performance of Li||Cu Cells and Li||Li Cells

**FIGURE 3:**
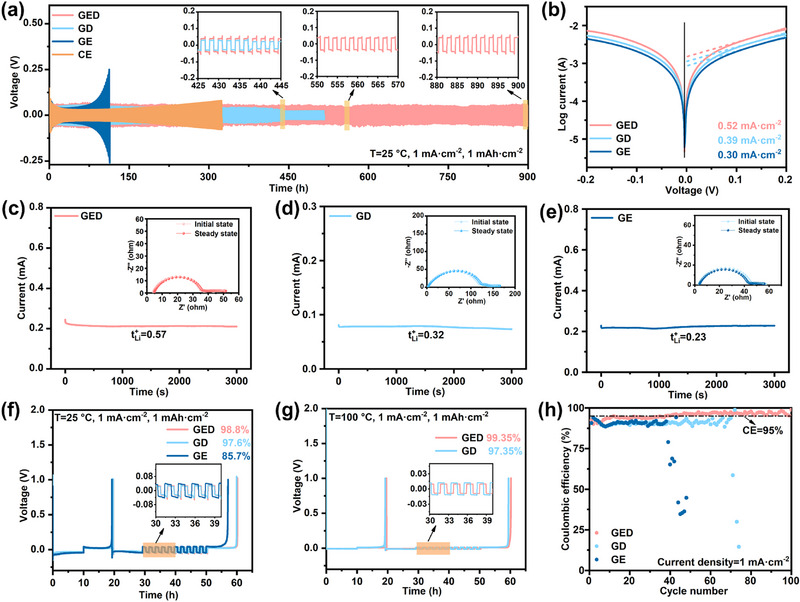
(a) Cyclic performance of Li||Li cells with different electrolytes at 25°C. (b) Tafel plots of Li||Li symmetric cells with different electrolytes. (c–e) Chronoamperometry for the Li^+^ transference number of various electrolytes. Average coulombic efficiency tests of Li||Cu cells with different electrolytes at (f) 25°C and (g) 100°C. (h) Long‐term cycling of Li||Cu half cells at a current density of 1 mA·cm^−2^ and capacity of 1 mAh·cm^−2^.

In LMBs using electrolytes with inappropriate solvation structures, they are usually accompanied by inhomogeneous lithium deposition and lithium dendrite growth, which eventually lead to continuous electrolyte depletion and the formation of non‐uniform SEI [28, 29]. To evaluate lithium compatibility across electrolytes, Li||Cu and Li||Li cells are used to assess lithium deposition/stripping behavior at varying temperatures. Long‐term cycling of Li||Li cells at 1 mA·cm^−^^2^ (Figure 3a) reveals stable operation for > 900 h with minimal voltage polarization in GED electrolyte. In contrast, CE and GE exhibit significant polarization voltage increase within 300 h, while GD experiences micro‐short circuits after 400 h. Furthermore, Tafel analysis (Figure 3b) demonstrates the highest exchange current density (0.52 mA·cm^−^^2^) for GED, indicating superior kinetics for uniform lithium deposition/stripping [30]. Chronoamperometry (Figure 3c–e) confirms GED's elevated Li^+^ transference number (t_Li_^+^ = 0.57), which mitigates concentration polarization during high‐rate discharge [31]. Consequently, GED achieves the lowest polarization voltages from 0.5 to 5 mA·cm^−^^2^ (Figure S20) versus comparative electrolytes.

**FIGURE 4:**
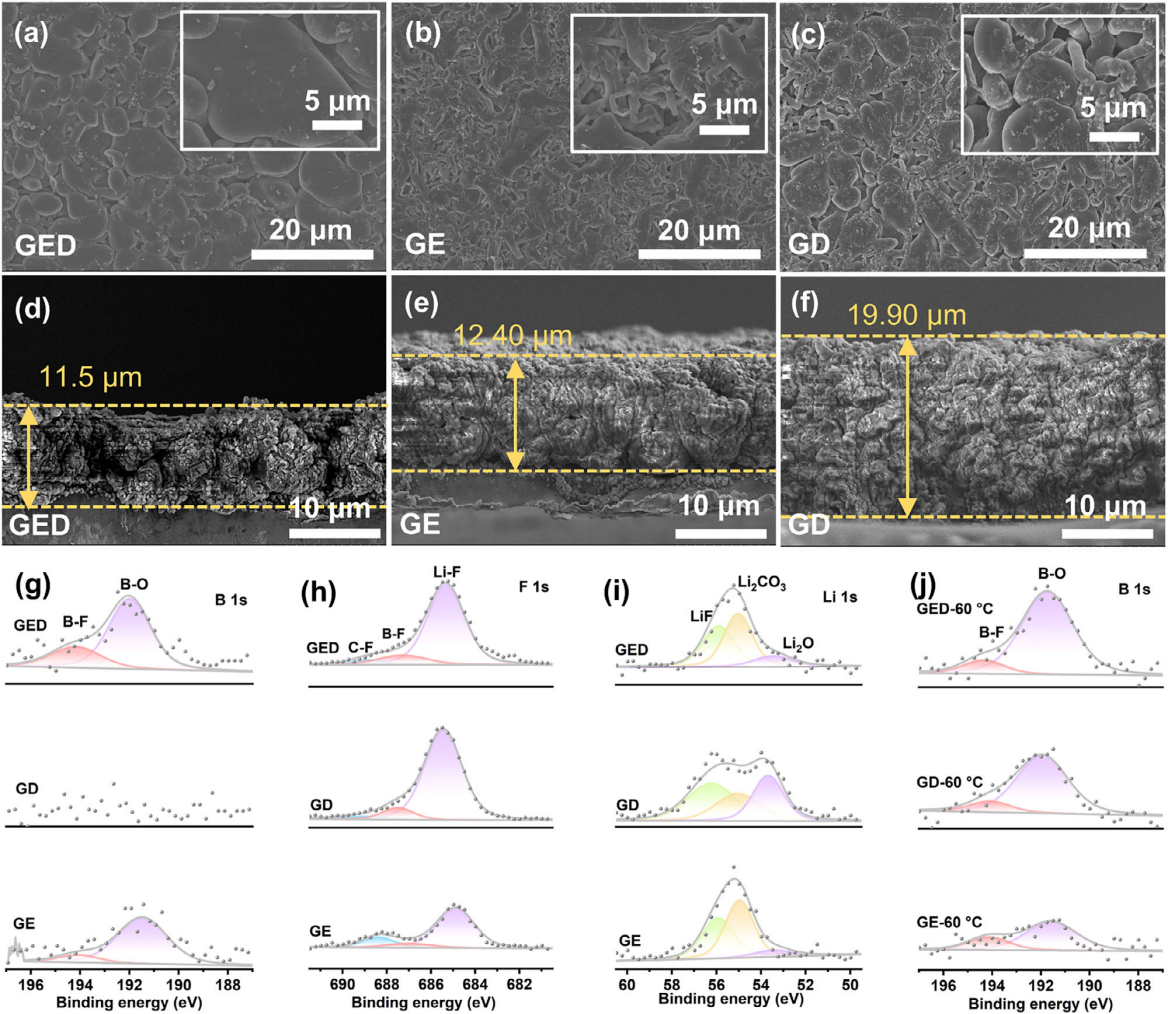
(a–c) SEM images illustrating Li deposition morphologies in Li||Cu cells with different electrolytes. (d,e) Cross‐sectional views of Li deposition morphologies in Li||Cu cells with different electrolytes. XPS investigation of (g) B 1s; (h) F 1s; (i) Li 1s of the Li metal surface with various electrolytes after 30 cycles. (j) XPS investigation of the Li metal surface with various electrolytes after 30 cycles at 60°C.

To further analyze continuous lithium deposition/stripping behavior, the coulombic efficiency of Li||Cu cells is quantified via the Aurbach method [32] across varying temperatures. As shown in Figure 3f,g, the GED electrolyte demonstrates an exceptional lithium affinity with Coulombic efficiency values reaching 98.8% at 25°C and 99.4% at 100°C. Even at low temperatures where lithium dendrites are extremely prone to growth (T = −20°C), it still maintains a high Coulombic efficiency of 92.80% (Figure S22), demonstrating excellent lithium metal compatibility over the entire temperature range. Moreover, as depicted in Figure 3h, the CE of the Li||Cu cells with GE and GD electrolytes exhibits a rapid decay below 95% within less than 70 cycles. In contrast, the GED electrolyte demonstrates relatively stable cycling performance. This behavior is attributed to the superior affinity between GED and lithium metal, which effectively inhibits the occurrence of parasitic reactions at the lithium metal interface. Furthermore, the morphology of lithium metal after deposition at different temperatures was analyzed using scanning electron microscopy (SEM) (with a total deposition amount of 2 mAh·cm^−^^2^). At room temperature (Figure 4a–f), GED yields compact and homogeneous lithium morphology with a deposit thickness of ≈ 11.5 µm, approaching the theoretical 10 µm value [33]. Critically, this dense uniformity persists at elevated temperatures (Figure S23), whereas GE and GD electrolytes exhibit progressive dendrite proliferation. Notably, GE generates granular/powdered deposits with heightened reactivity—accelerating dead lithium formation, dendritic growth, and thermal runaway risks via separator penetration. These findings collectively validate our electrolyte design in enabling uniform lithium deposition, dendrite suppression, and interfacial stability under extreme thermal conditions.

X‐ray photoelectron spectroscopy (XPS) analysis of room‐temperature cycled lithium metal anodes reveals the SEI composition. For GED, stronger B‐O and B‐F signals in B 1s spectrum confirm an enhanced ODFB^−^ participation in primary solvation shells (Figure 4g). GE exhibits weaker yet detectable boron‐related signals than GD due to greater anion involvement, consistent with prior MD simulations. Li 1s and F 1s spectra demonstrate intensified inorganic components (LiF, Li_2_CO_3_) in GED (Figure 4h,i). This inorganic‐rich SEI suppresses lithium‐electrolyte side reactions, enhances ion transport, and promotes uniform lithium deposition [34]. This also explains the exceptional performance of the GED‐based cells in both Li||Li and Li||Cu cell configurations. B 1s analysis at 60°C confirms the persistence of strong signals in GED (Figure 4j), evidencing ODFB^−^’s critical role in high‐temperature interphase formation. Reduced organic components in C 1s and O 1s spectra demonstrate the inorganic‐rich SEI's dual function (Figure S25): inhibiting solvent decomposition and exhibiting superior thermal stability at elevated temperatures.

### 2.3 Performance of Li||NCM811 Cells and Application Performance

**FIGURE 5:**
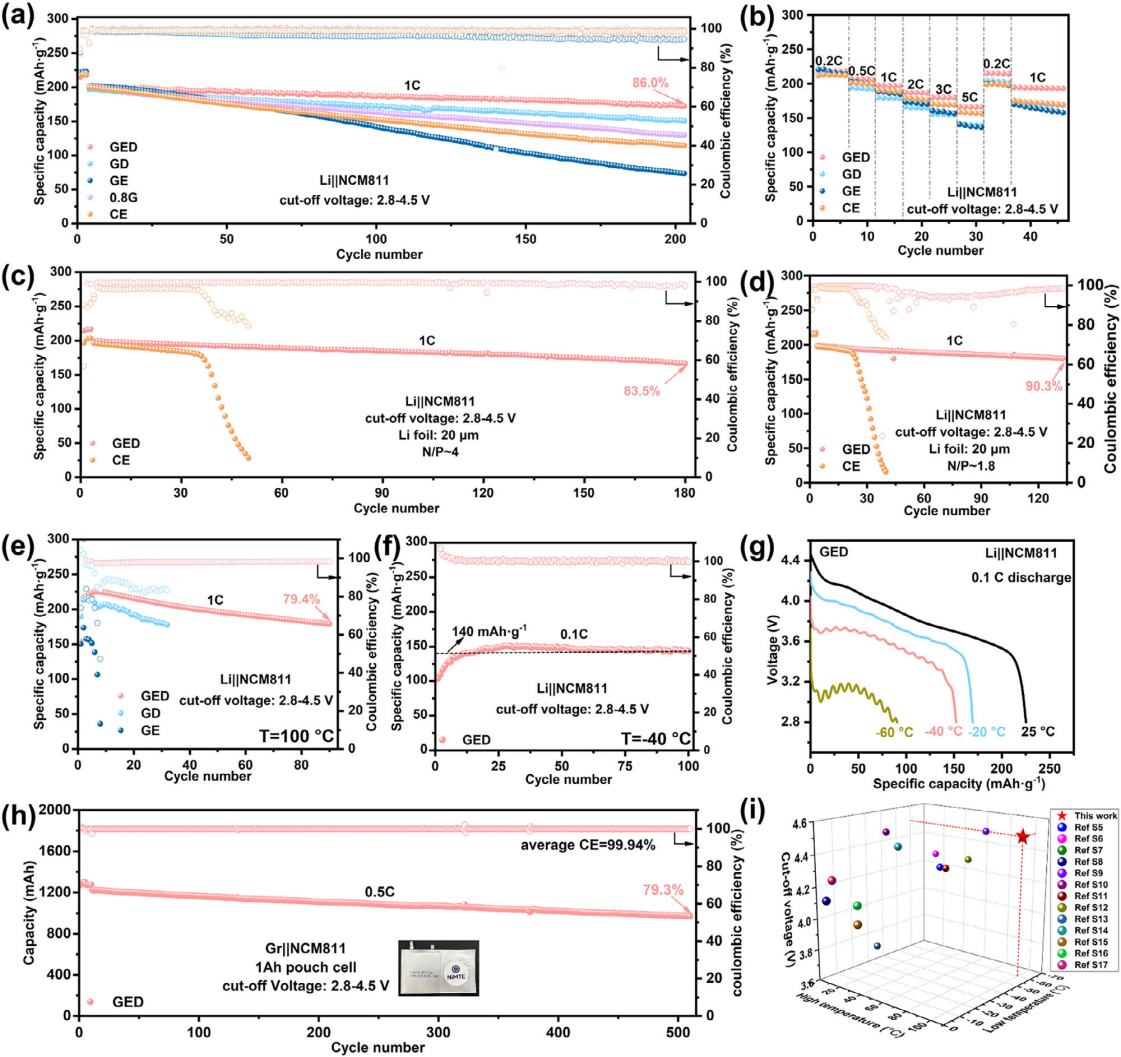
(a) Cycle performance of Li||NCM811 half cells with different electrolytes at 25°C. (b) Rate performance of Li||NCM811 half cells with different electrolytes. Cycling performance of the Li||NCM811 full‐cell using CE and GED electrolytes (c) N/P ratio ≈ 4; (d) N/P ratio ≈ 1.8. (e) Cyclic performance of Li||NCM811 half cells with different electrolytes at 100°C. (f) Cyclic performance of Li||NCM811 half cells with GED electrolyte at −40°C. (g) Discharge curves of Li||NCM811 cells with GED electrolyte at different temperatures. (h) Cycle performance of 1 Ah pouch cell with GED electrolyte. (i) Operating temperature and cut‐off voltage of the cells are based on the GED electrolyte and previous reported electrolytes.

At elevated temperatures, battery failure primarily stems from cathode structural degradation, underscoring the critical importance of electrolyte‐mediated cathode protection [35]. To evaluate GED electrolyte compatibility with high‐nickel cathodes, Li||NCM811 cells underwent cycling tests and characterization across temperatures. As shown in Figure 5a, GED demonstrates superior capacity retention (86% after 200 cycles at 4.5 V cutoff) versus GE, GD, 0.8G, and CE electrolytes under ambient conditions, confirming that the robust SEI derived from the GED electrolyte can effectively protect the cathode structure and mitigate continuous electrolyte consumption. Supporting charge/discharge curves and dQ/dV analyses corroborate this finding (Figures S26 and S27). Rate capability tests (Figure 5b) reveal GED delivers 166.3 mAh·g^−^^1^ at 5C, benefiting from its suitable ionic conductivity and low viscosity. Practical applicability was further validated using Li foil (20 µm)||NCM811 cells (N/P ≈ 4). GED maintains 83.5% capacity retention after 180 cycles (Figure 5c), while CE exhibits rapid capacity decay after 30 cycles due to limited lithium inventory. Under more stringent conditions (N/P ≈ 1.8), GED‐based cells achieve 91.3% capacity retention after 130 cycles (Figure 5d).

Cycling performance of Li||NCM811 cells with different electrolytes under extreme temperatures was tested. Benefiting from the inorganic‐rich stable interphase enabled by anion‐dominated solvation and GVL's high flash point, Li||NCM811 cell with GED electrolyte achieves cycle stably over 200 cycles at 60°C (Figure S28), maintains 80.1% after 100 cycles at 80°C (Figure S29), and delivers 178.8 mAh·g^−^^1^ with high average coulombic efficiency after 90 cycles at 100°C (Figure 5e). At low temperatures, enhanced desolvation kinetics and ion transport enable GED to exhibit near‐zero capacity fade over 200 cycles at −20°C (0.1C; Figure S34) with average discharge capacity exceeding 170 mAh·g^−^^1^ (75% of room‐temperature capacity). It sustains approximately 62% room‐temperature capacity after 100 cycles at −40°C (0.1C, Figure 5f) while other electrolytes fail to operate at −40°C. Notably, even at an extremely low temperature of −60°C, the cell with GED electrolyte can still deliver a discharge capacity of 90.4 mAh·g^−^^1^ at 0.1C (Figure 5g). Moreover, the 1 Ah pouch cell of Gr||NCM811 based on GED electrolyte exhibits a capacity retention rate of 79.3% after 500 cycles at a rate of 0.5 C, with an average coulombic efficiency as high as 99.94% (Figure 5h). It can be seen that the GED electrolyte has good practical application performance. These results confirm GED's operational capability across −60°C to 100°C, surpassing most reported wide‐temperature LMB electrolytes in both temperature range and cycling stability (Figure 5i; Table S2). Furthermore, EIS with DRT transformation of cycled cells demonstrates consistently lower R_EEI_ for GED across all tested temperatures, confirming superior interfacial stability under extreme temperatures (Figure S36a–f).

**FIGURE 6:**
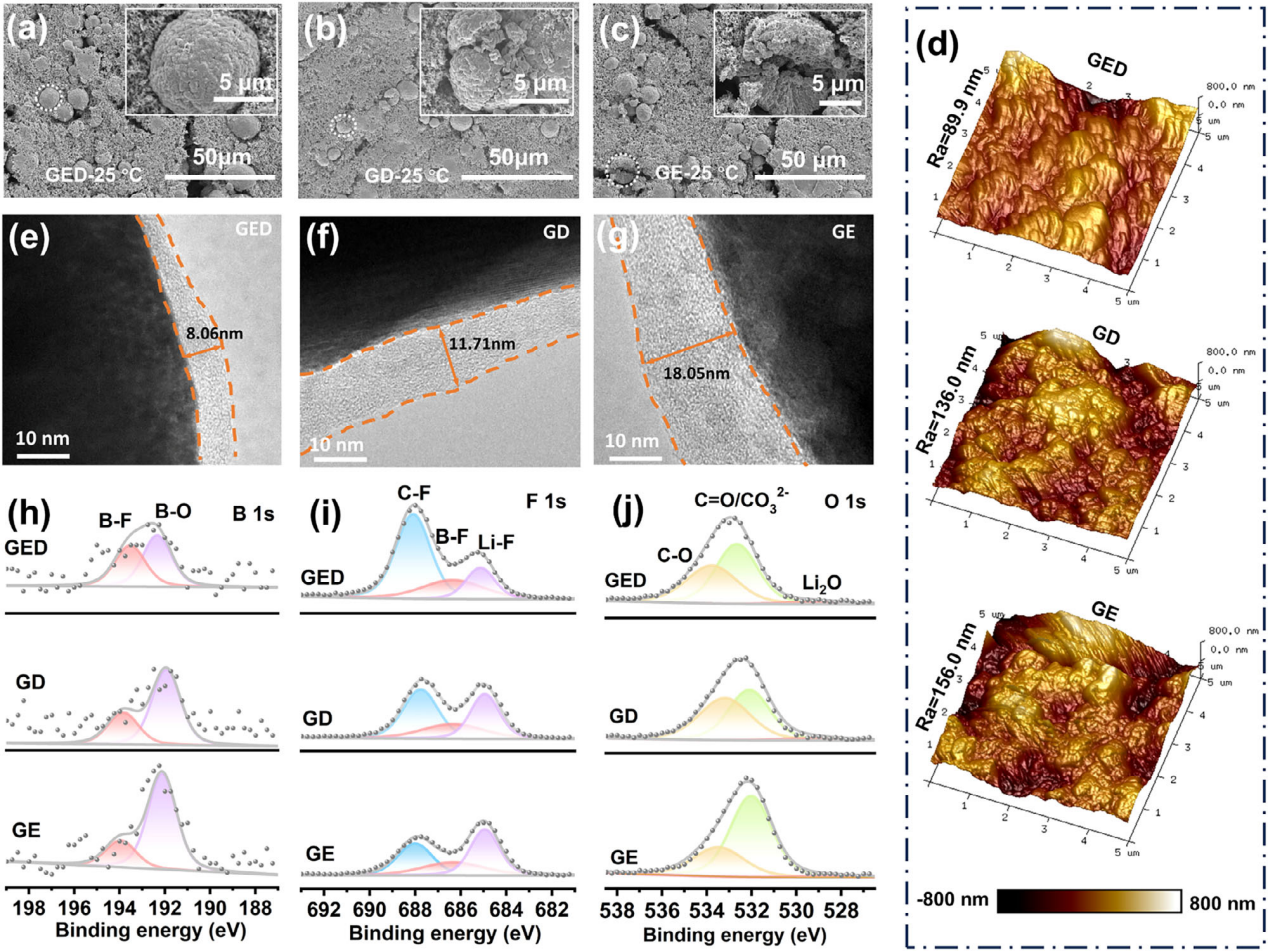
(a–c) SEM images of cycled NCM811 electrodes with different electrolytes after 200 cycles. (d) AFM images of cycled NCM811 electrodes with different electrolytes after 200 cycles. (e–g) TEM images of cycled NCM811 electrodes with different electrolytes after 50 cycles. XPS investigation of (h) B 1s; (i) F 1s; (j) O 1s of the NCM811 surface with various electrolytes after 30 cycles.

The surface morphology of the cycled NCM811 electrodes is analyzed using SEM and transmission electron microscopy (TEM). As shown in Figure 6a–c, the cathode particles of GED‐based cells remain relatively intact after cycling, while those of electrodes with other electrolytes exhibit varying degrees of fragmentation, demonstrating the protective effect of GED electrolyte on the cathode. As depicted in Figure 6e–g, the GED electrolyte forms the thinnest and most uniform CEI layer with a thickness of approximately 8.06 nm, whereas the other two electrolytes lead to the formation of thick and uneven CEI due to side reactions with the cathode. Moreover, at high temperatures, various side reactions on the cathode side are exacerbated, making structural collapse more likely. However, the electrode sheets of GED‐based batteries still maintain a good morphology, which is attributed to the effective protection of the CEI layer (Figure S40). Atomic force microscopy (AFM) tests on the roughness of cycled NCM811 electrode sheets also confirm that the GED electrolyte promotes the formation of a more uniform and denser CEI (Figure 6d). Compared with the electrode sheets cycled with the other two electrolytes, the GED sample maintains a lower roughness level (Ra = 89.9 nm).

XPS analysis of CEI composition after room‐temperature and 60°C cycling reveals GED electrolyte's cathode protection mechanism. B 1s results (Figure 6h; Figure S43a) demonstrate consistently enhanced B‐F signals for GED at both temperatures, confirming increased ODFB^−^ involvement in cathode interphase formation. Appropriate LiF signals in F 1s (Figure 6i; Figure S43b) spectra primarily result from B‐F bond cleavage in decomposed ODFB^−^. Reduced organic components in O 1s/C 1s spectra (Figure 6j; Figure S42) reflect a suppressed solvent decomposition by GED's solvation structure. The results of O 1s and C 1s (Figure S43c,d) of the cathode after cycling at 60°C show that the organic components in the CEI of GED remain at a low level. Such a CEI rich in inorganic components can greatly improve its thermal stability at high temperatures and provide certain protection for the structure of high‐nickel cathodes [36, 37]. XRD analysis (Figure S44) validates these findings showing minimal low‐angle shift of NCM811's (003) peak in GED compared to significant shifts in controls confirming superior structural integrity preservation [38, 39].

## 3 Conclusion

This work pioneers the application of cyclic carboxylate‐based electrolytes in lithium metal batteries by introducing weak solvents with different coordination abilities. Leveraging their competitive solvation effects and solvent‐solvent interactions (van der Waals forces), we successfully engineer an anion‐dominated loose solvation structure with fast desolvation kinetics, achieving an ultra‐wide operating temperature range of from −60°C to 100°C. Through MD calculations as well as characterization methods such as infrared spectroscopy and Raman spectroscopy, we demonstrate that DFEC and ETFA promote greater ODFB^−^ coordination while displacing high‐solvation‐energy GVL from the primary solvation shell. XPS analyses confirm this solvation design facilitates thermally stable EEI that preserves cathode structural integrity and enables rapid desolvation kinetics for homogeneous lithium deposition. The resulting electrolyte delivers exceptional performance: stable cycling at 4.5 V cutoff under extreme conditions (100°C at 1 C and −40°C at 0.1 C), 90.4 mAh·g^−^^1^ capacity at −60°C, and 90.3% capacity retention after 130 cycles under stringent conditions (N/P ≈ 1.8). This study provides fundamental insights for using cyclic carboxylates in lithium metal batteries and establishes a new design paradigm for extreme‐condition electrolytes.

## Conflicts of Interest

The authors declare no conflicts of interest.

## Supporting information




**Supporting File**: advs74265‐sup‐0001‐SuppMat.pdf.

## Data Availability

The data that support the findings of this study are available from the corresponding author upon reasonable request.;
