# Supplementary material for: γ‐Valerolactone‐Based Anion‐Dominated Loose Solvation Electrolyte Enables Stable Lithium Metal Batteries from −60°C to 100°C
Source: Adv Sci (Weinh). 2026 Feb 6;13(22):e23560. doi: 10.1002/advs.202523560 (PMC13088308; doi:10.1002/advs.202523560)
Supplement: Supplementary file 1 — Supporting File: advs74265‐sup‐0001‐SuppMat.pdf. [file ADVS-13-e23560-s001.pdf]

Supporting Information

γ-Valerolactone-Based Anion-Dominated Loose Solvation Electrolyte Enables Stable Lithium Metal Batteries from −60 °C to 100 °C

Lei Zhang, Tianle Zheng*, Qing Ming, Keyu Zheng, Jin Zhu, Yiyao Xiao, Said Amzil, Mengqi Wu, Shengyao Luo, Meilan Peng, Yinghui Li, Xiuxia Zuo, Peter Müller-Buschbaum*, Ya-Jun Cheng*, and Yonggao Xia*

L. Zhang, Q. Ming, S. Luo
School of Materials Science and Chemical Engineering, Ningbo University, Ningbo 315211, Zhejiang, P. R. China

L. Zhang, Q. Ming, Y. Xiao, S. Amzil, M. Wu, S. Luo, M. Peng, Y. Li, K. Zheng, J. Zhu, X. Zuo, Y.-J. Cheng, Y. Xia
Ningbo Institute of Materials Technology and Engineering, Chinese Academy of Sciences, 1219 Zhongguan West Rd, Ningbo 315201, Zhejiang, P. R. China
E-mail: chengyj@nimte.ac.cn, xiayg@nimte.ac.cn

Y.-J. Cheng
College of Renewable Energy, Hohai University, 1915 Hohai Ave, Changzhou 213200, Jiangsu, P. R. China

T. Zheng, P. Müller-Buschbaum
Technical University of Munich, TUM School of Natural Sciences, Department of Physics, Chair for Functional Materials, 85748 Garching, Germany
E-mail: tianle.zheng@ph.tum.de, muellerb@ph.tum.de

T. Zheng
Jiangsu Cnano Technology Co., Ltd., Zhenjiang 212000, Jiangsu, P. R. China

X. Zuo
College of New Energy, Ningbo University of Technology, Ningbo, 315336, PR China

Y. Xia
Center of Materials Science and Optoelectronics Engineering, University of Chinese Academy of Sciences, 19A Yuquan Rd, Shijingshan District, Beijing 100049, P. R. China

Experimental Section

Materials: γ-valerolactone (GVL, 98%) and ethyl trifluoroacetate (ETFA, 99%) were purchased from Aladdin and Macklin, respectively. Difluoro ethylene carbonate (DFEC, 99.99%) was purchased from Zhuhai Saiwei. The commercial electrolyte was purchased from Jiangsu Guotaihuarong. Cathode electrodes were prepared by mixing $LiNi_{0.8}Co_{0.1}Mn_{0.1}O_2$ (NCM811, Zhejiang Ronbay), conductive carbon black (Super P, Timcal) and polyvinylidene fluoride (PVDF, Solvay) (mass ratio = 80:10:10) with N-methyl pyrrolidone (NMP, 99%, Aladdin) as the diluent. The cathode slurries were cast onto an aluminium current collector (99.5%, Huizhoulianhe) and dried at 110 °C for 12 h under vacuum. Finally, the electrodes were cut into a circular shape with a diameter of 14 mm. The mass loading of anodes was 3.8-4.2 $mg\cdot cm^{-2}$ (half cell and full cell with N/P ratio ≈ 4) and 8-8.5 $mg\cdot cm^{-2}$ (full cell with N/P ratio ≈ 1.8). Lithium foil (99.9%, 450 μm and 20 μm in thickness) was purchased from China Energy Lithium Co., Ltd. Li||NCM811 cells were assembled with Whatman separators (GF/F 1825) and 160 μL electrolyte in coin cells (CR 2032-type). The Li||Li and Li||Cu cells were fabricated using procedures identical to those described above. The 1 Ah Gr||NCM811 (loading: 15.1 $mg\cdot cm^{-2}$) pouch cell was purchased from Dongguan Kelude Laboratory Equipment Technology Co., Ltd.

Electrochemical Measurements: The 2032 coin-type cells were assembled in an argon-filled glove box (O_2 and H_2O < 0.01 ppm). Different cells with various electrolytes were tested on the LAND cell test system (Wuhan LAND Electronics). Li||NCM811 cells were activated for 3 cycles at 0.2 C, followed by 1 C cycling in the voltage interval of 2.8-4.5 V to evaluate the high-voltage stability of the electrolyte and all batteries subjected to high- and low-temperature cycling were activated at room temperature. The oxidation/reduction resistance of the electrolytes was measured using linear scanning voltammetry (LSV), which was carried out on an electrochemical workstation (CHI760E). The measurements employed a voltage scanning range from 2

V to 6 V and a scanning speed of 0.5 $mV\cdot s^{-1}$. The Electrochemical Impedance Spectroscopy (EIS, Solartron Analytical 1470E) test was conducted within the frequency range of 0.01 Hz to 100 kHz. The in-situ EIS testing method involves assembling a Li||NCM811 battery and measuring the EIS every 0.1 V within the voltage window of 2.8-4.5 V. The computation of the distribution of relaxation time (DRT) method is based on Bayesian ridge regression (also known as Tikhonov regularization). The exchange current density was calculated based on the Tafel equation, measured at a scan rate of 0.1 $mV\cdot s^{-1}$ from -0.2 to 0.2 V. The Li^+ transference number ($t_{Li}{}^+$) was measured under a polarization voltage of 10 mV using Li||Li cells. Both tests were also carried out on the electrochemical workstation (CHI760E). The $t_{Li}{}^+$ was s calculated using the equation:[1]

$$t_{Li^+} = \frac{I^S(\Delta V - I^0 R^0)}{I^0(\Delta V - I^S R^S)} \qquad (1)$$

Where I^0 and I^S are the initial current and steady-state current during the polarization process, respectively. R^0 and R^S are the resistances of the Li||Li battery before and after the measurement of the tested battery.

Furthermore, the GITT test was mainly conducted after the activation of the Li||NCM811 battery. The battery was charged at a rate of 0.2 C for 5 minutes to reach a stable state (E_S). The charging-resting process was repeated until the cutoff voltage of 4.5 V was reached, and then the battery was discharged to 2.8 V under the same conditions. The Li^+ coefficient in the cathode can be calculated using the equation that follows Fick's second law, and the equation is finally simplified as follows:[2]

$$D_{Li^+} = \frac{4}{\pi\tau}\left[\frac{n_m V_m}{S}\right]^2\left[\frac{\Delta E_s}{\Delta E_t}\right]^2 \qquad (2)$$

Where τ is the relaxation time (3600 s), n_m is the molar amount of the active material, V_m is the molar volume of the active material (20.56 $cm^3\cdot mol^{-1}$), and S is the electrode/electrolyte contact area (1.54 cm^2).

Cyclic voltammetry (CV, Solartron Analytical 1470E) tests was performed on Li||NCM811 cells. The tests employed a potential window of 2.8 to 4.5 V (vs. Li^+/Li) at a scan rate of 0.1 $mV\cdot s^{-1}$.

Characterization: Differential Scanning Calorimetry (DSC, NETZSCH) testing was

employed to investigate the thermodynamic behavior of the electrolyte at low temperatures, with the test procedure involving cooling from room temperature to −150 °C at a rate of 10 °C per minute. The solvation structure of different electrolytes was characterized by infrared spectroscopy (IR, Cary660+620) and nuclear magnetic resonance (NMR, AVANCE III 400MHz). The surface morphology of electrodes after cycling was examined by transmission electron microscope (TEM, JEOL2100), scanning electron microscope (SEM, Hitachi S4800) and atomic force microscope (AFM, Dimension ICON). Additionally, the components of the SEI and CEI layers were examined by X-ray photoelectron spectroscopy (XPS, AXIS Ultra DLD). X-ray diffraction (XRD, Bruker D8 Advance) was employed to examine the structure of the cycled electrode materials.

Theoretical calculation methods: The density functional theory (DFT) method was employed for quantum chemical calculations using the Material Studio 2023 software. A hybrid method utilizing the Lee-Yang-Parr correlation functional (B3LYP) in the Dmol3 module was employed.[3] All molecules were modeled within a simulated solvent environment (conductor-like screening model, COSMO) with a chosen solvent (acetone). The binding energy of Li^+-solvent/anion was calculated as follows:[4]

$$E_{binding} = E_{Li^+} + E_{solvent} - E_{complex} \qquad (3)$$

Where $E_{binding}$ is the binding energy of Li^+ and solvent, E_{Li^+}, $E_{complex}$ are the electron energy of single Li^+ and Li^+-solvent complex, respectively.

Material Dynamic (MD) simulations were performed utilizing the Forcite module. 0.8G electrolyte consisted 100 Li^+, 100 $ODFB^-$ and 1320 GVL; GE electrolyte consisted 100 Li^+, 100 $ODFB^-$, 660 GVL and 525 ETFA; GD electrolyte consisted 100 Li^+, 100 $ODFB^-$, 1055 GVL and 245 DFEC; GED electrolyte consisted 100 Li^+, 100 $ODFB^-$, 395 GVL, 525 ETFA and 245 DFEC. All molecular dynamic simulations were calculated with the COMPASSIII force field, utilizing a 1.0 fs (femtosecond) constant time step. To achieve the desired equilibrium condition, the systems first undergoes geometric optimization and annealing steps to reach the energy-minimized frame, and then the systems underwent equilibration steps in the NPT ensemble using the

Berendsen barostat for at least 200 ps. The barostat maintained a pressure of 0.1 GPa with a decay constant of 0.1 ps. Then the production runs were conducted in the NVT ensemble for a period of 2 ns (nanosecond). A Nosé thermostat operating at a temperature of 298/373 K was used for all the processes. The length of the simulation was adequate to achieve equilibrium in the electrolyte systems.

The HOMO, LUMO energies and electrostatic potential calculations were performed as follows: For individual solvents or lithium salts, the structures were optimized using the hybrid/B3LYP method before proceeding with the calculations. For different electrolyte solvation structures, representative solvation structures were extracted from MD simulation boxes after NPT and NVT processes, and then calculations were conducted on them using the aforementioned optimization method.

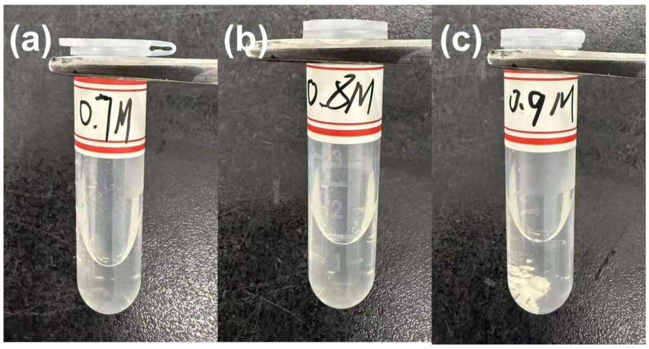

Figure S1. Dissolution status of lithium salts at varying concentrations (a-c).

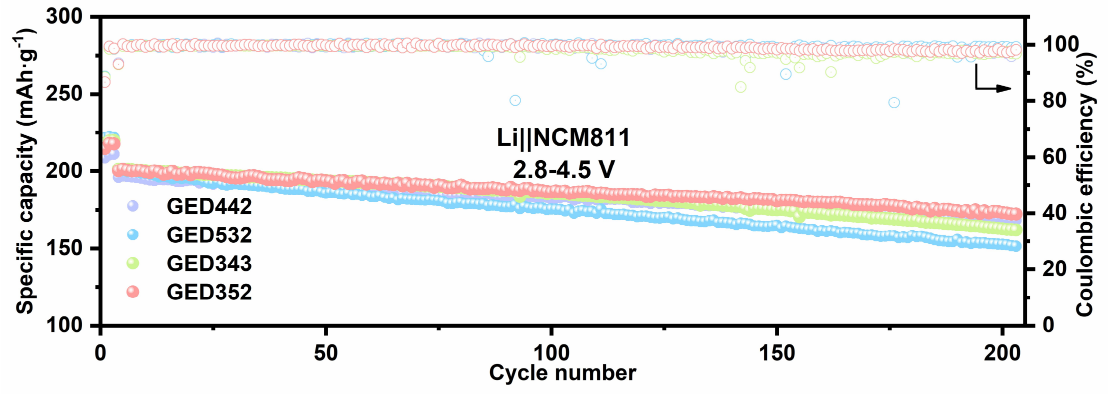

Figure S2. Cycling performance of three solvents at different ratios.

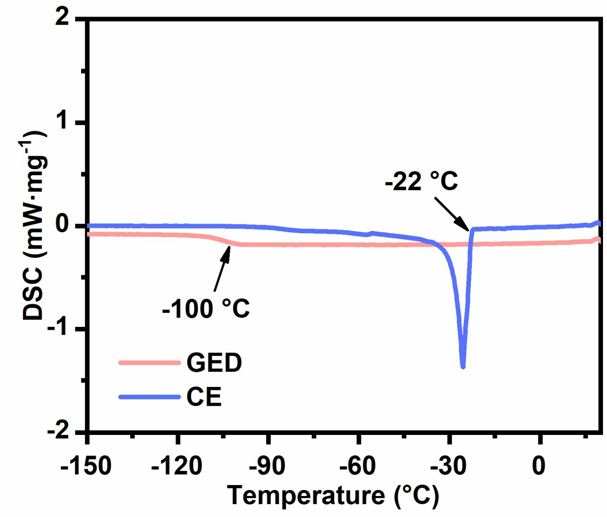

Figure S3. DSC curves of CE and GED electrolytes.

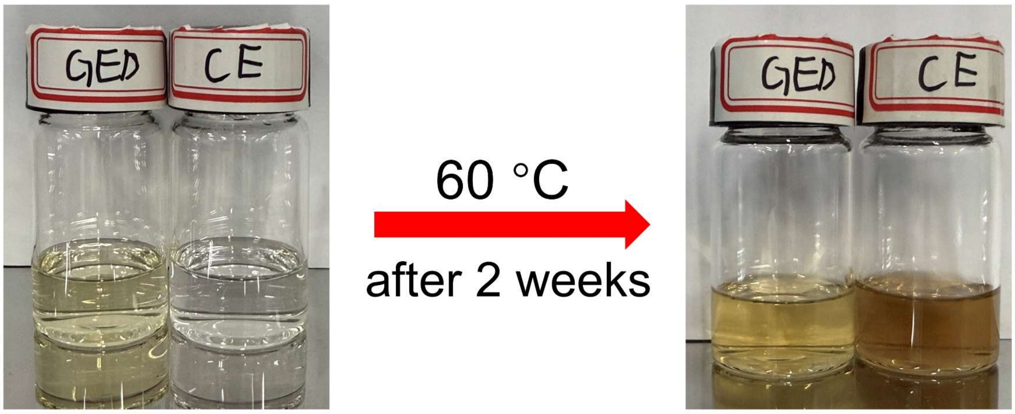

Figure S4. State figures of GED and CE electrolytes after two weeks of storage (T=60 °C).

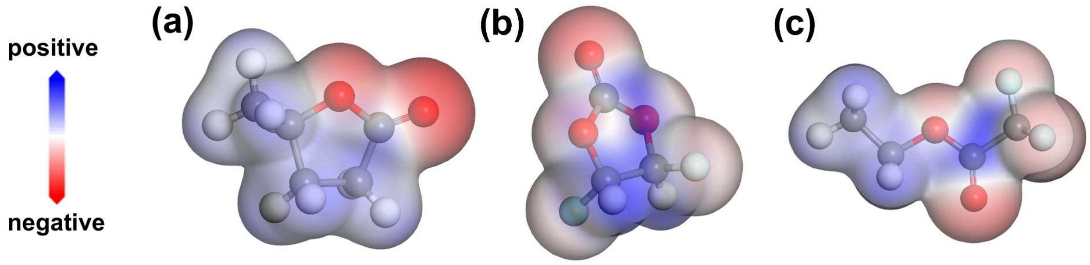

Figure S5. Electrostatic potential mappings of GVL, DEFC, ETFA based on the electron density (a-c). Red: O; Grey: C; Blue: F; White: H; Purple: Li.

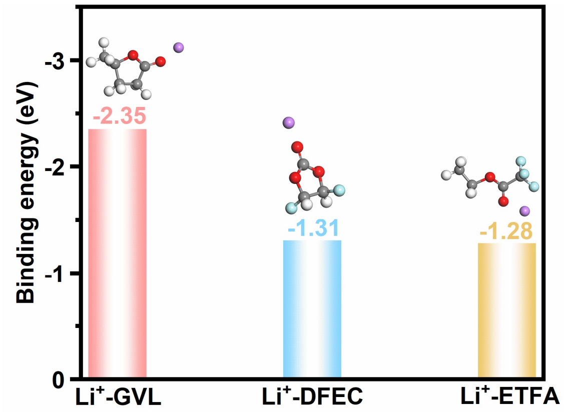

Figure S6. Binding energy of Li^+ with various solvents calculated via DFT.

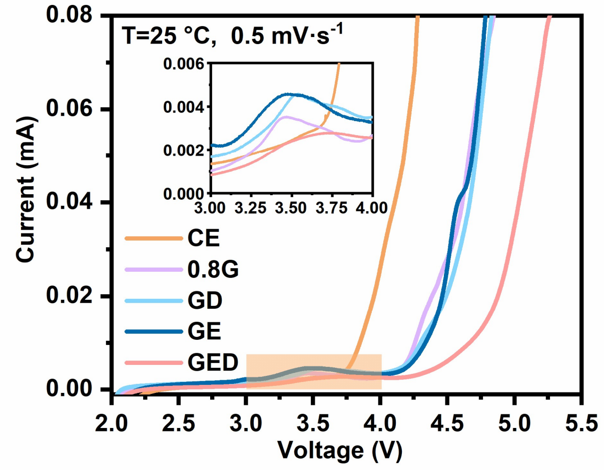

Figure S7. Li||steel positive LSV curves of various electrolytes (T=25 ℃).

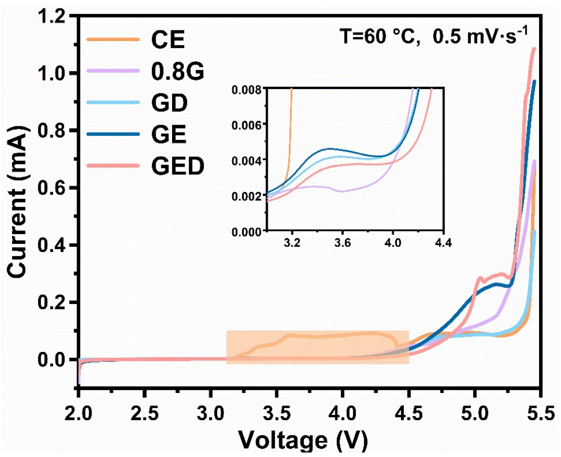

Figure S8. Li||steel positive LSV curves of various electrolytes (T=60 ℃).

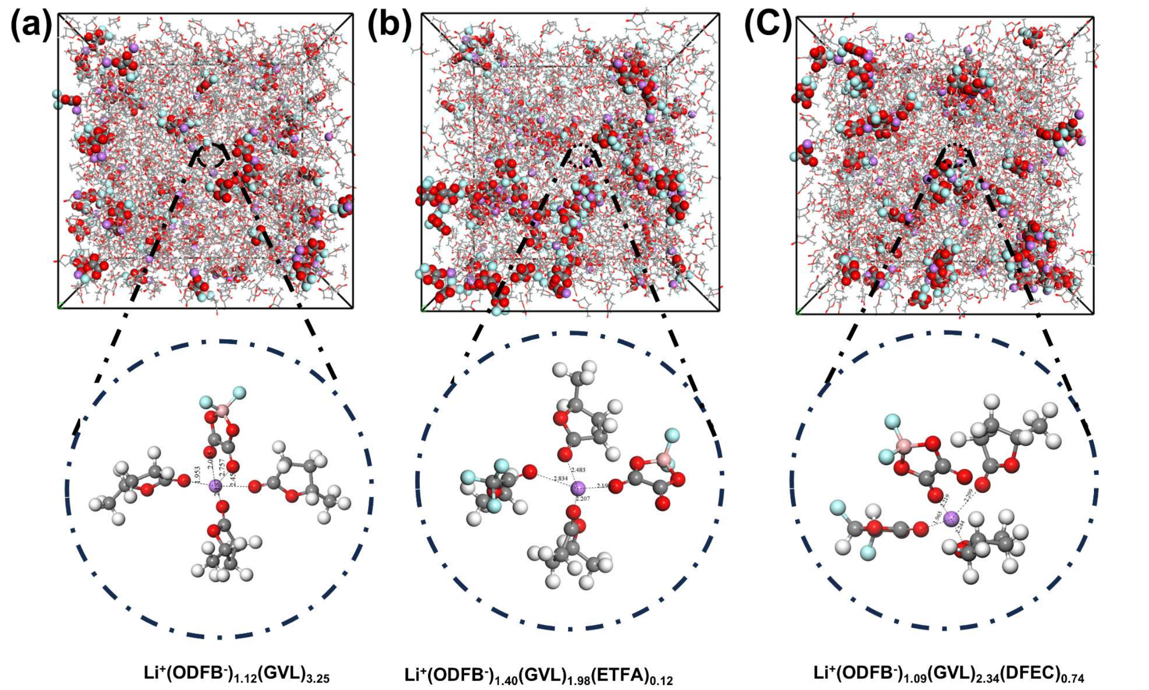

Figure S9. MD simulation box and representative solvation structures extracted from MD simulation boxes of 0.8G (a), GE (b) and GD (c) electrolytes at 25 °C. Pink: B.

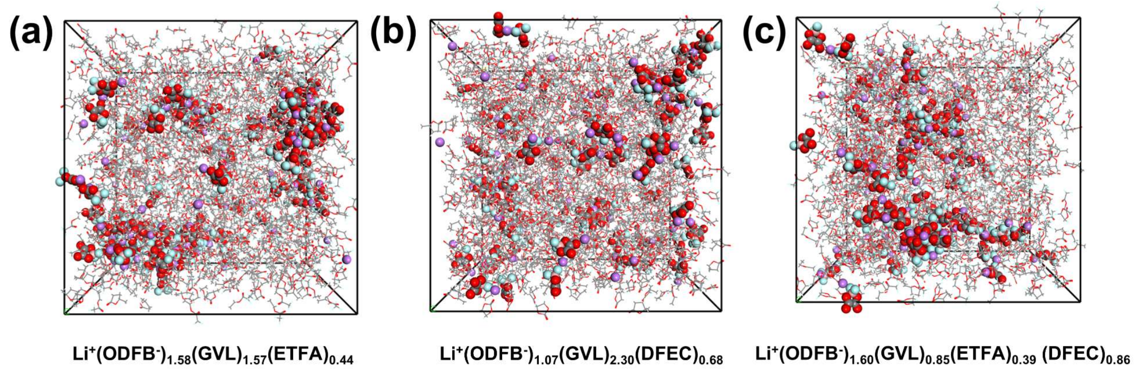

Figure S10. MD simulation box of GE (a), GD (b) and GED (c) electrolytes at 100 °C.

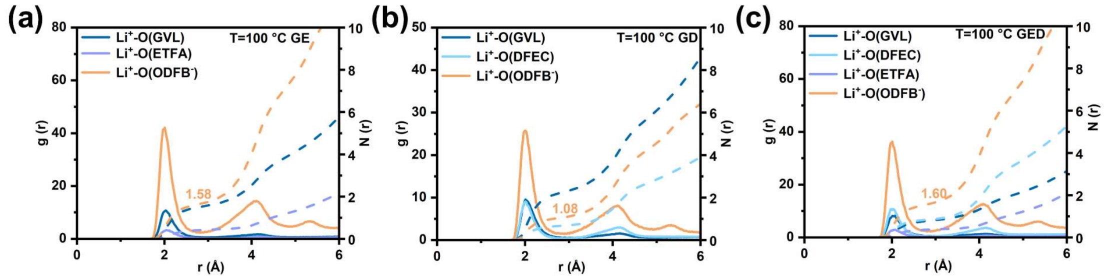

Figure S11. Radial distribution function and coordinated number of different electrolytes at 100 °C.

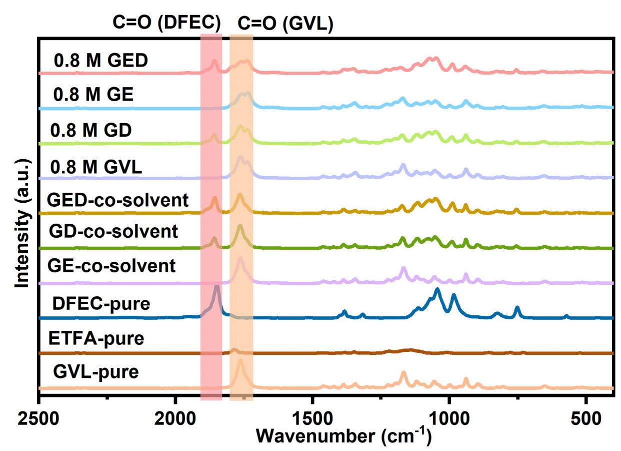

Figure S12. FTIR spectra of pure solvents and electrolytes.

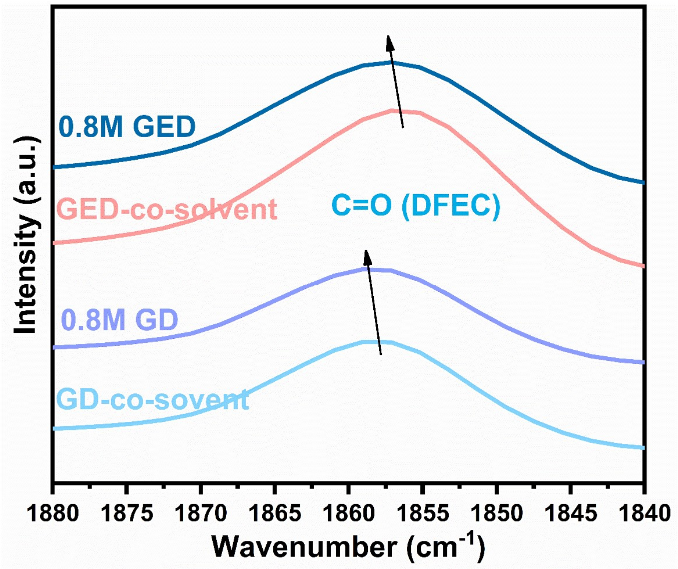

Figure S13. FTIR spectra of C=O in DFEC within various mixed solvents and electrolytes.

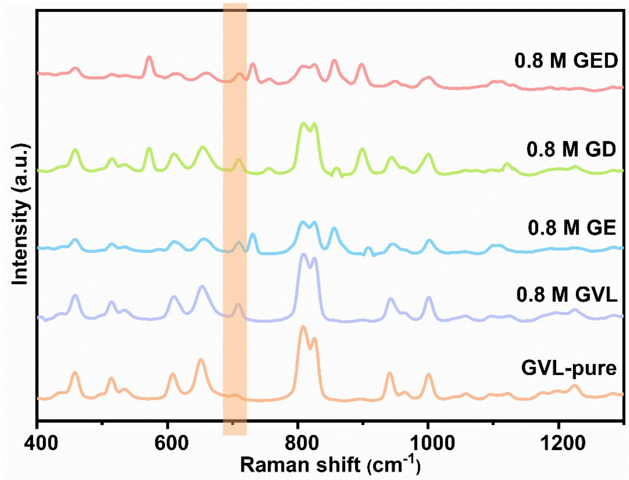

Figure S14. Raman spectra of pure GVL and various electrolytes.

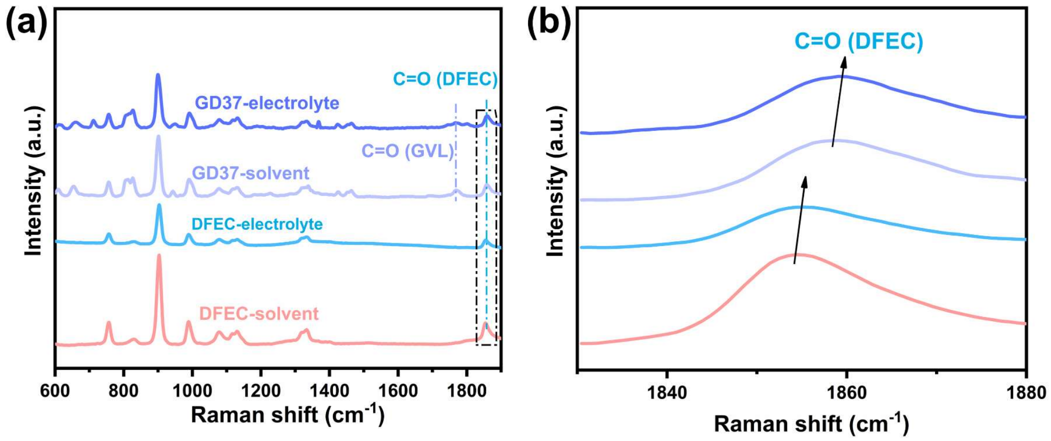

Figure S15. Raman spectra of various solvents and various electrolytes (a)600-1900 cm^{-1}; (b)1830-1880 cm^{-1}.

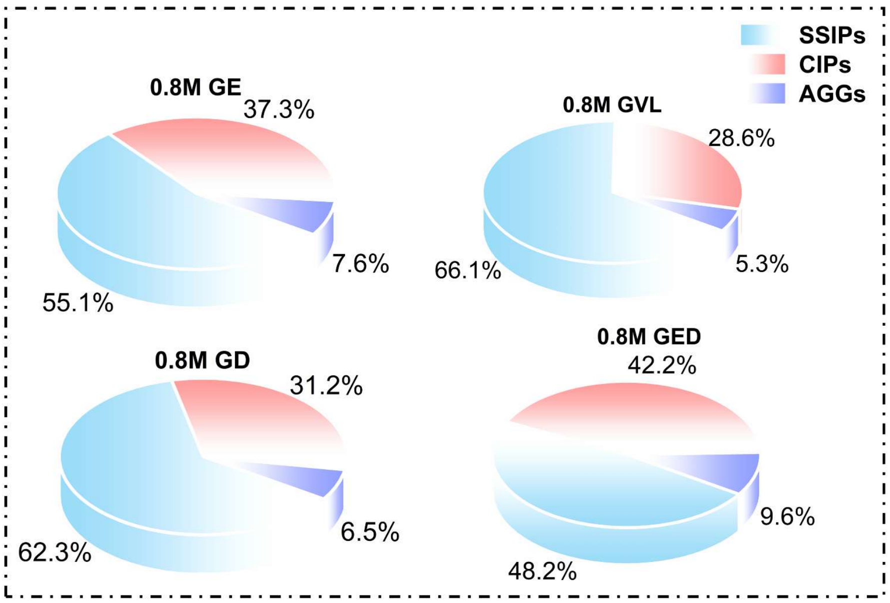

Figure S16. Relative compositions of the three states of $ODFB^{-}$ in various electrolytes were determined based on the Raman spectra.

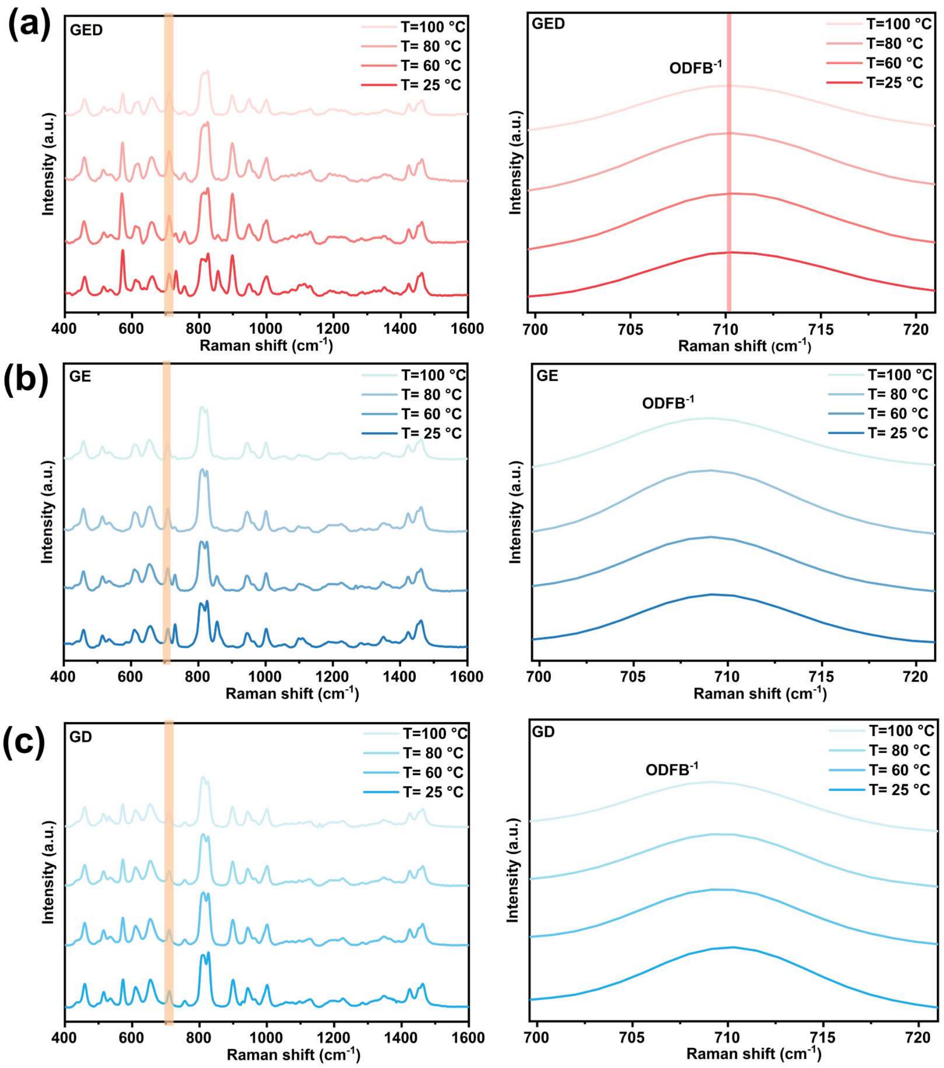

Figure S17. Variable-temperature Raman spectra of GED, GE and GD (a-c).

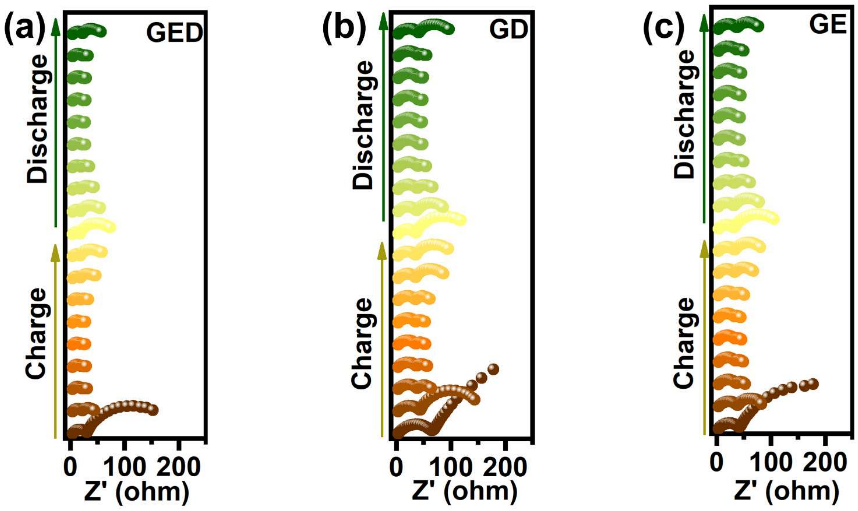

Figure S18. In-situ EIS analysis of Li||NCM811 Cells with various electrolyte.

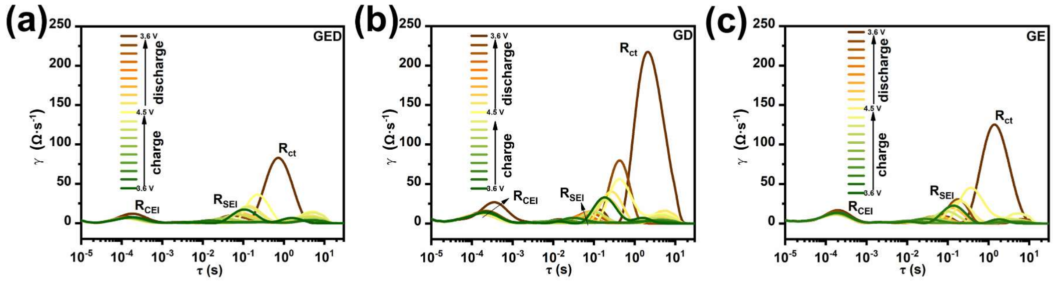

Figure S19. DRT results with different electrolytes (a-c).

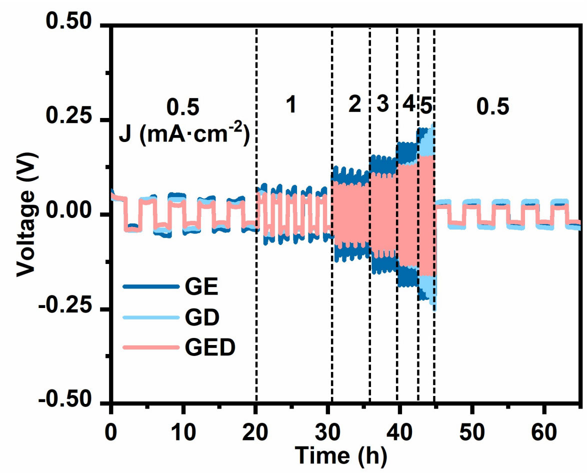

Figure S20. Rate performances of the symmetrical Li||Li cells with GE, GD and GED electrolytes at different current densities.

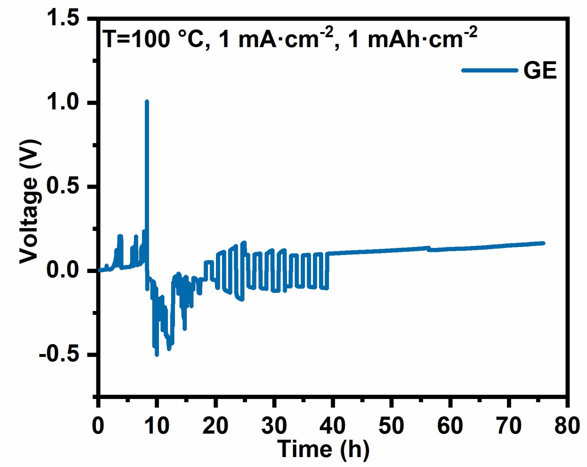

Figure S21. GE's electrochemical performance of Li||Cu at 100 °C.

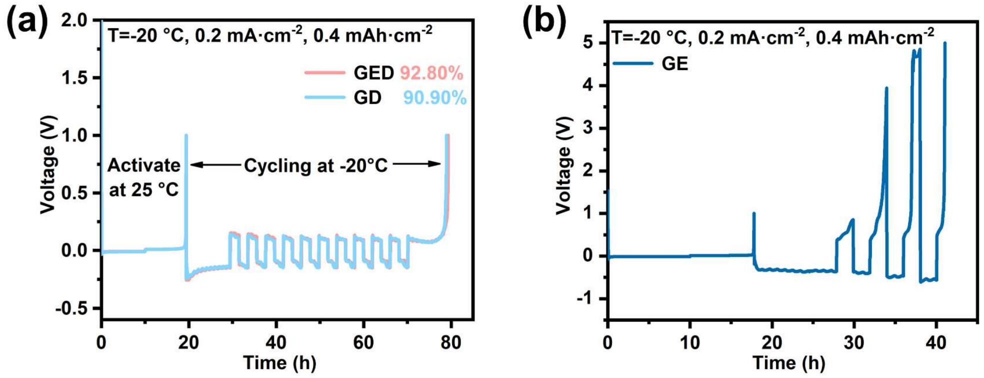

Figure S22. Electrochemical performance of Li||Cu with (a) GED and GD electrolytes; (b) GE electrolyte at -20 ℃.

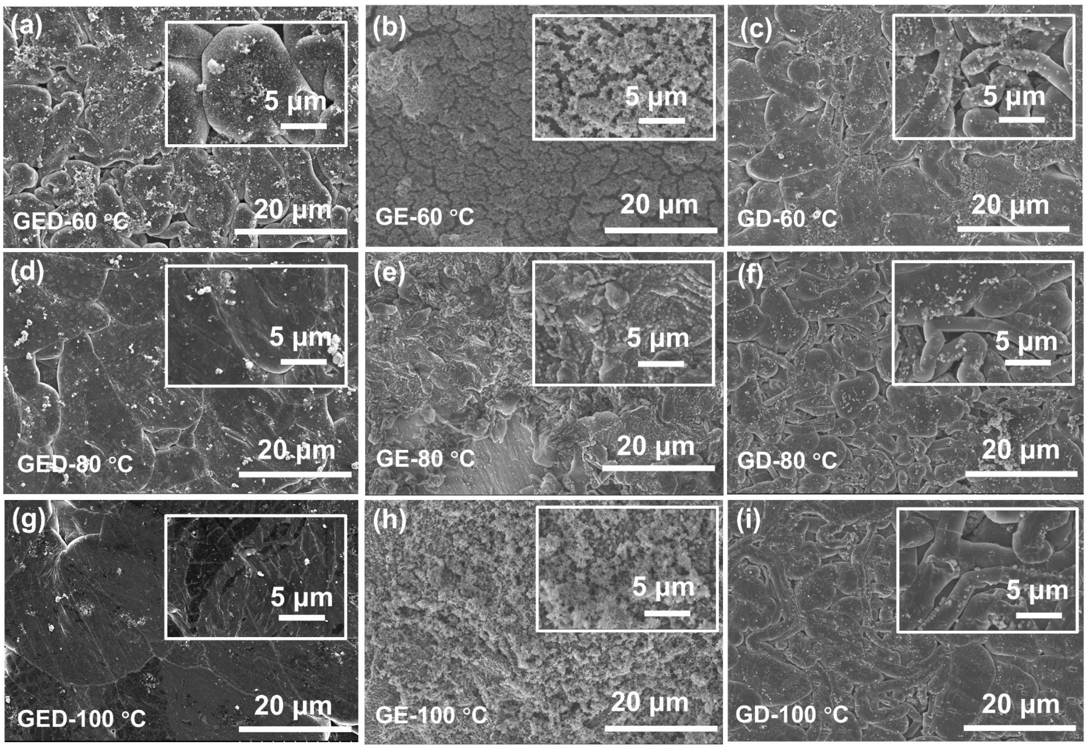

Figure S23. SEM images illustrating Li deposition morphologies in Li||Cu cells with different electrolytes under an areal capacity of 2 mAh·cm^{-2} at high temperature (a-i).

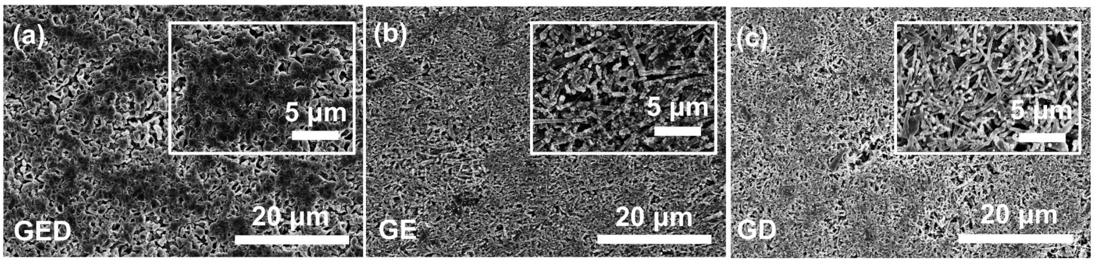

Figure S24. SEM images illustrating Li deposition morphologies in Li||Cu cells with different electrolytes at -20 ℃ temperature (a-c).

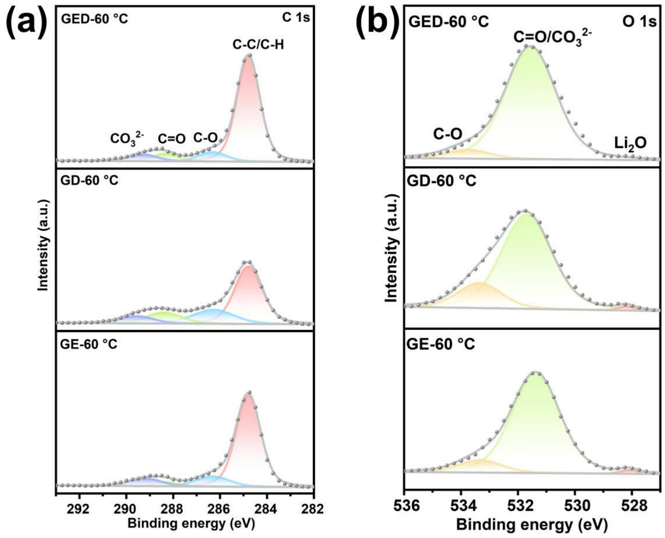

Figure S25. XPS investigation of (a) C 1s and (b) O 1s of the Li metal surface with various electrolytes after 30 cycles at 60 ℃.

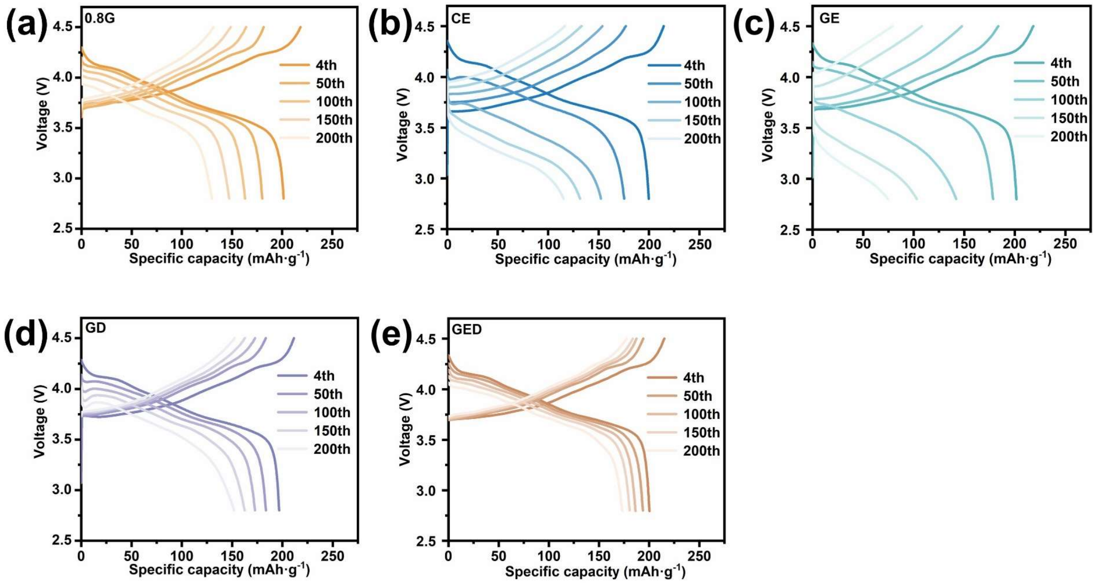

Figure S26. Charge–discharge voltage profiles of the Li||NCM811 with various electrolytes (a-e).

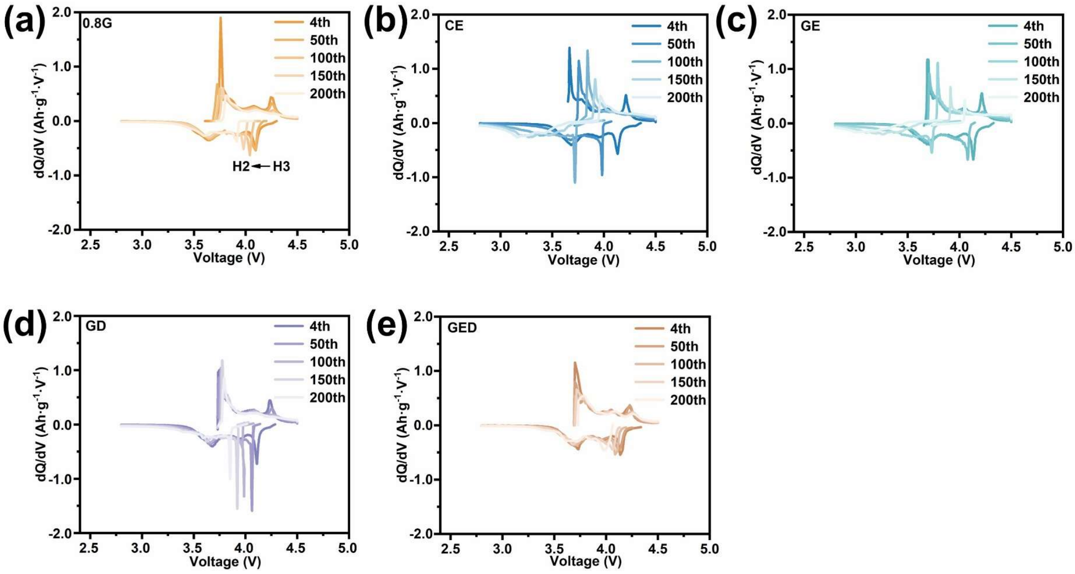

Figure S27. DQ/dV curves of the Li||NCM811 with various electrolytes (a-e).

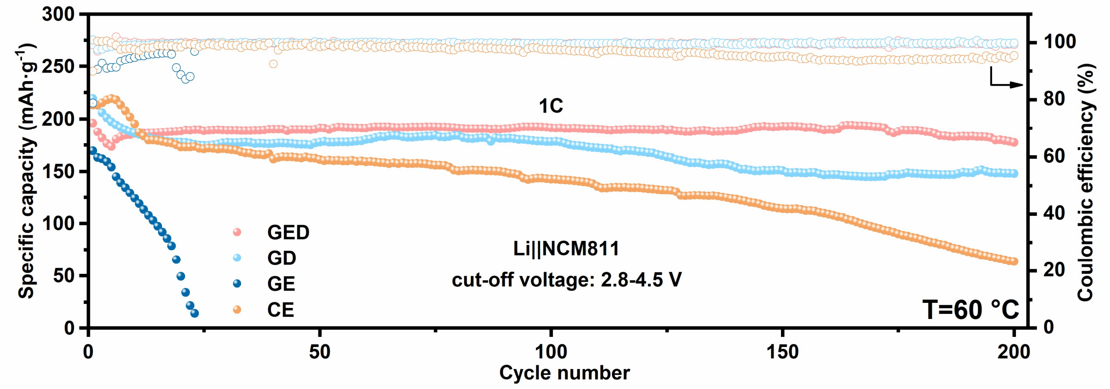

Figure S28. Cyclic performance of Li||NCM811 half cells with various electrolytes at 60 ℃.

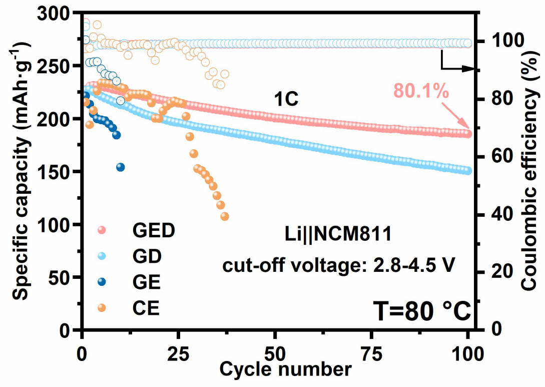

Figure S29. Cyclic performance of Li||NCM811 half cells with various electrolytes at -20 ℃.

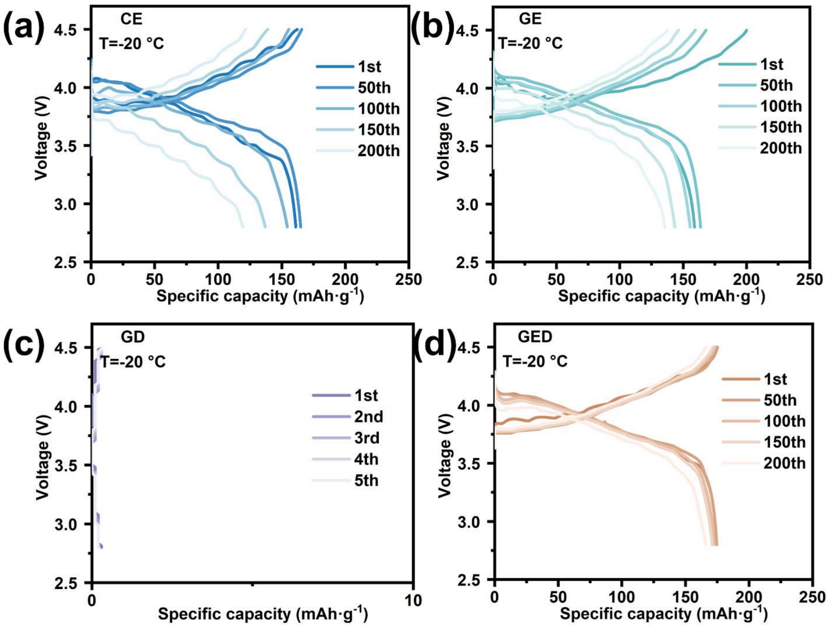

Figure S30. Charge–discharge voltage profiles of the Li||NCM811 with various electrolytes at -20 ℃ (a-e).

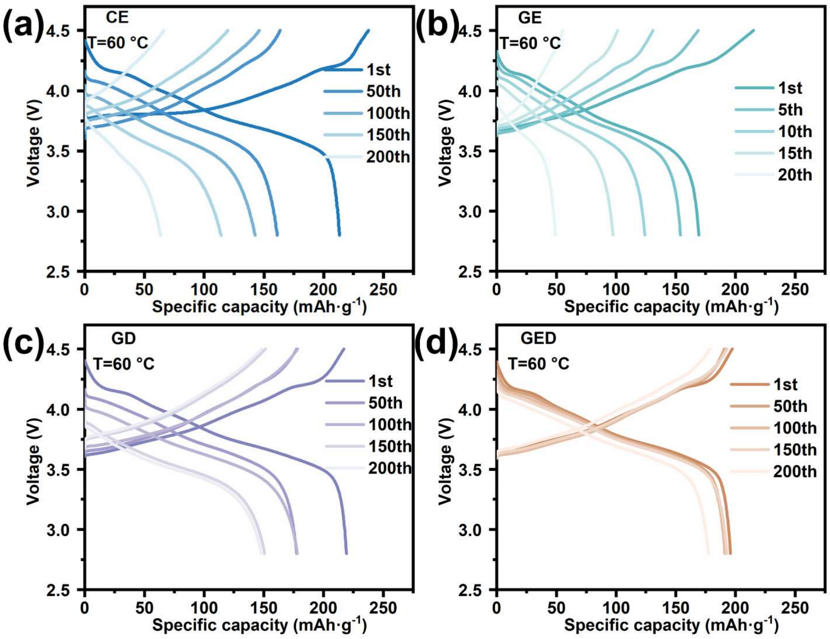

Figure S31. Charge–discharge voltage profiles of the Li||NCM811 with various electrolytes at 60 ℃ (a-e).

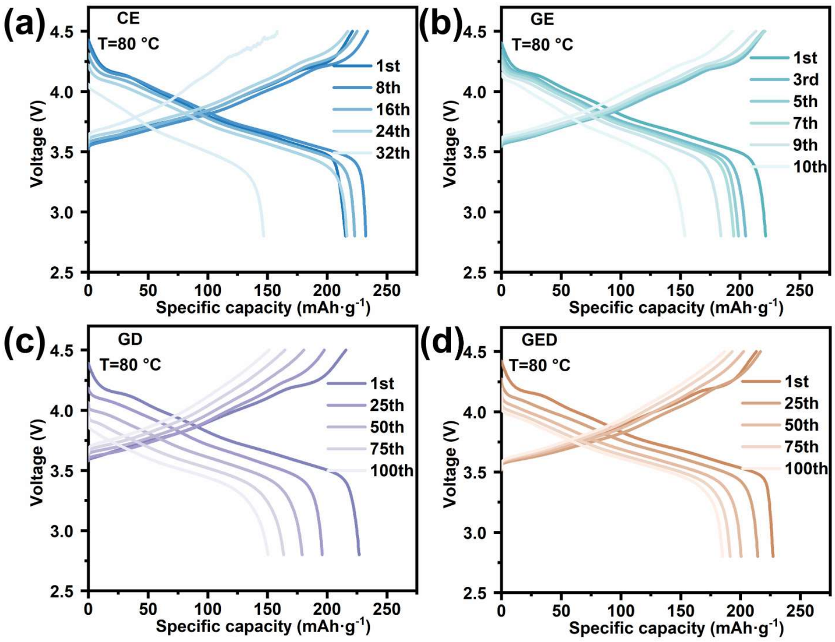

Figure S32. Charge–discharge voltage profiles of the Li||NCM811 with various electrolytes at 80 °C (a-e).

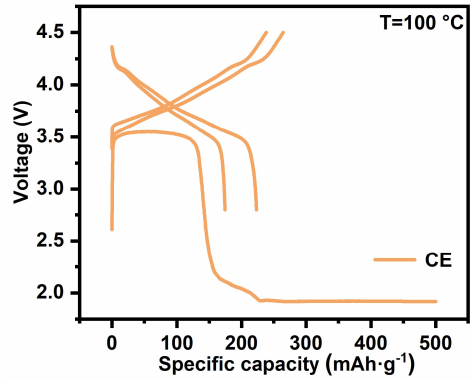

Figure S33. Charge–discharge voltage profiles of the Li||NCM811 with CE electrolyte at 100 °C.

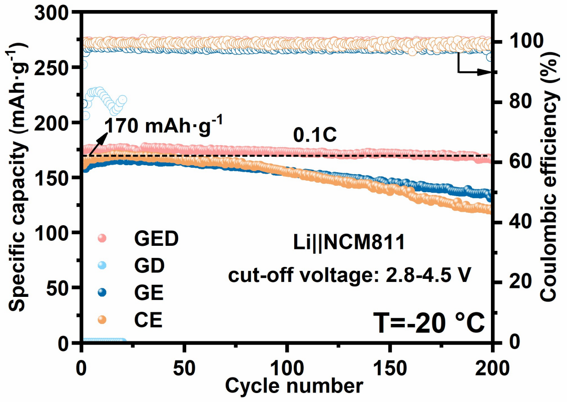

Figure S34. Cyclic performance of Li||NCM811 half cells with various electrolytes at -20 °C.

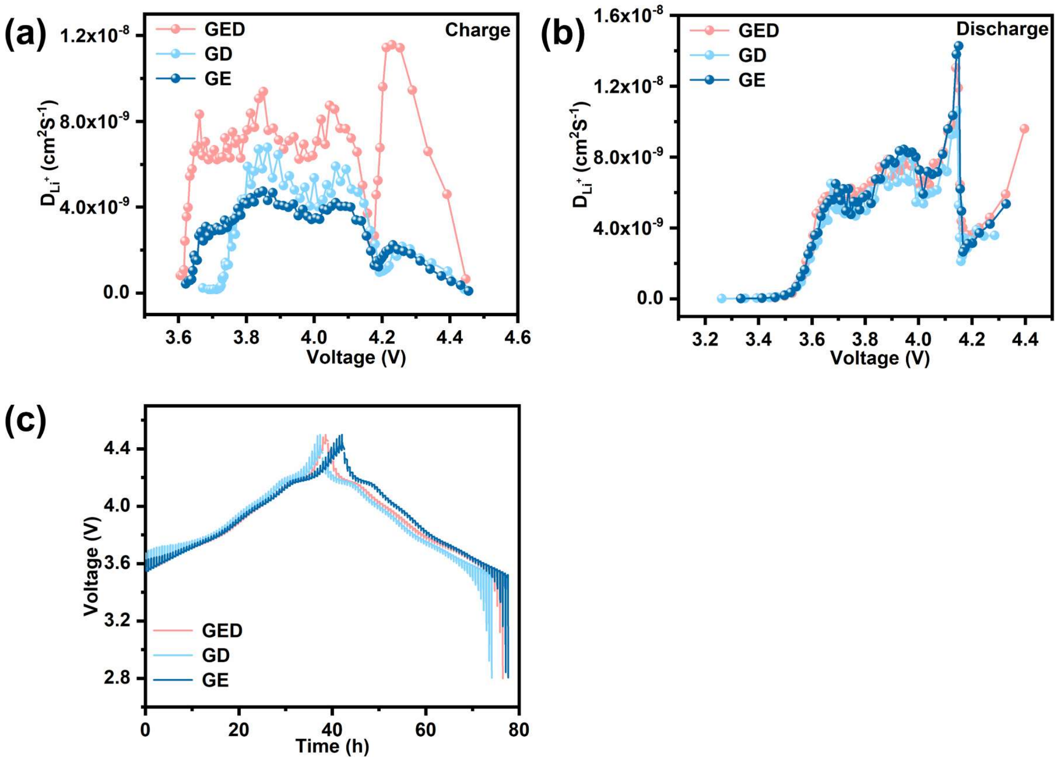

Figure S35. GITT analysis of Li||NCM811 half cells with GED, GD, and GE electrolytes (a,b); GITT curves of Li||NCM811 half cells (c).

Note: The diffusion on the cathode side of different electrolyte systems was investigated using the galvanostatic intermittent titration technique (GITT). It can be found that the diffusion coefficient of GED during the charging process is much higher than that of the other two samples, representing its faster ion transport performance in the CEI.

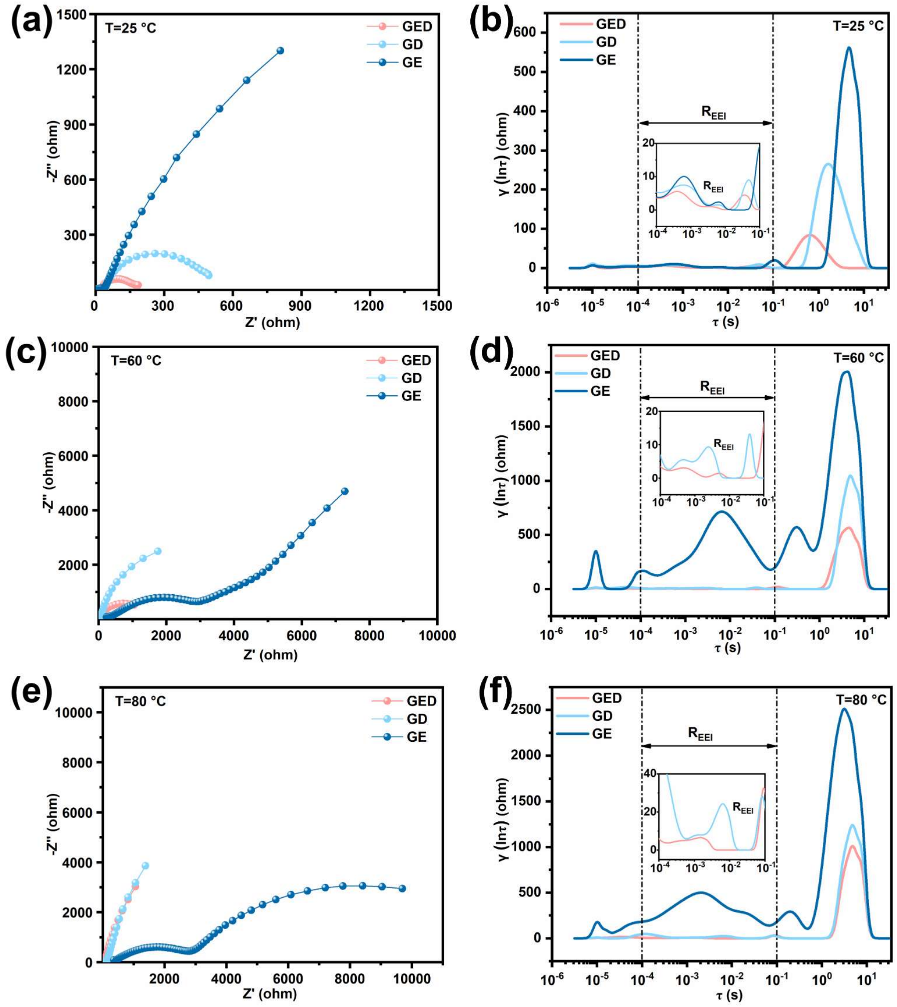

Figure S36. EIS of Li||NCM811 half cells with GED, GD and GE electrolytes after cycle at 25 ℃, 60 ℃, 80 ℃ (a,c,e); DRT results of Li||NCM811 half cells with GED, GD, and GE electrolytes after cycle at 25 ℃, 60 ℃, 80 ℃ (b,d,f).

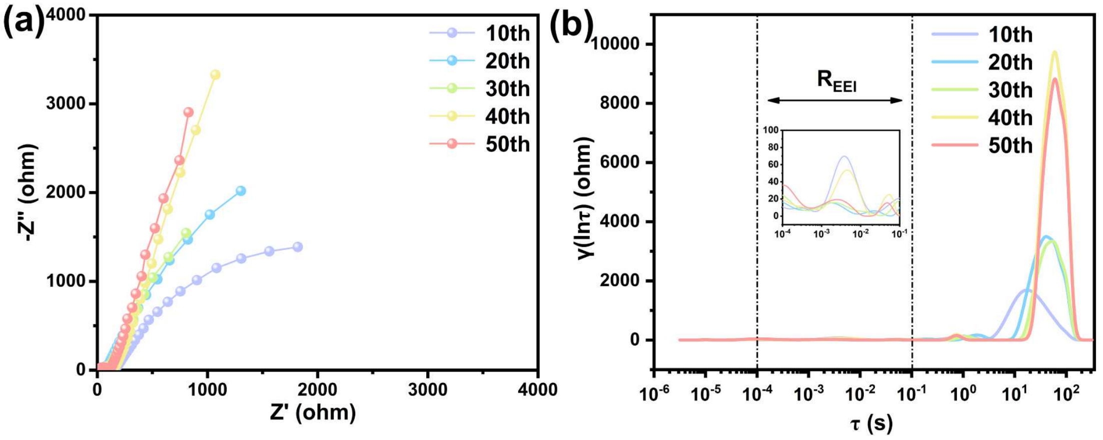

Figure S37. EIS of Li||NCM811 cell with GED electrolyte after different cycles at 100 °C (a); DRT results of Li||NCM811 cell with GED electrolyte after different cycles at 100 °C (b).

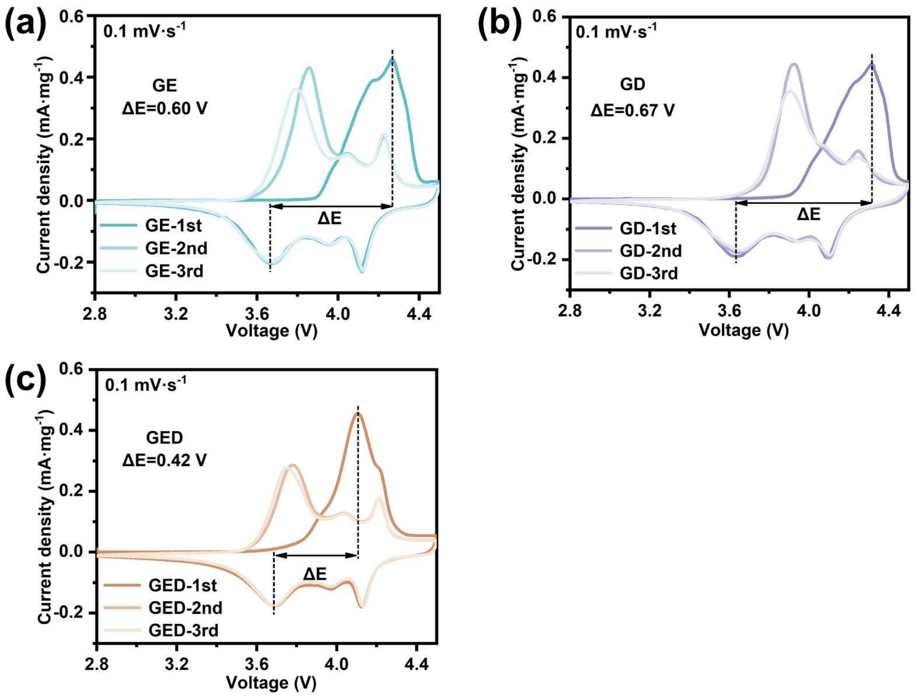

Figure S38. CV curves of the Li||NCM811 half cells in various electrolytes with the scan rate of 0.1 $mV \cdot s^{-1}$ (a-c).

Note: Cyclic Voltammetry (CV) tests reveal that the Li||NCM811 cell with GED electrolyte exhibits a smaller redox peak separation ($\Delta V = 0.42$ V) at a scan rate of 0.1 $mV \cdot s^{-1}$, indicating lower polarization of the electrolyte.

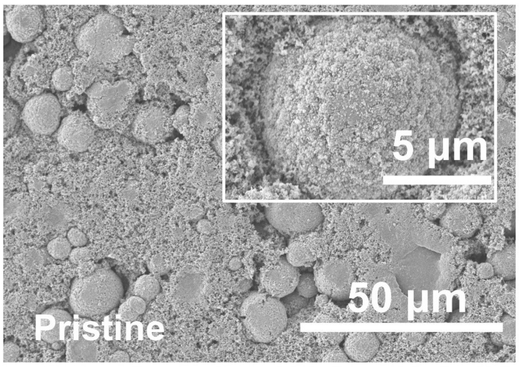

Figure S39. SEM image of pristine NCM811 electrode.

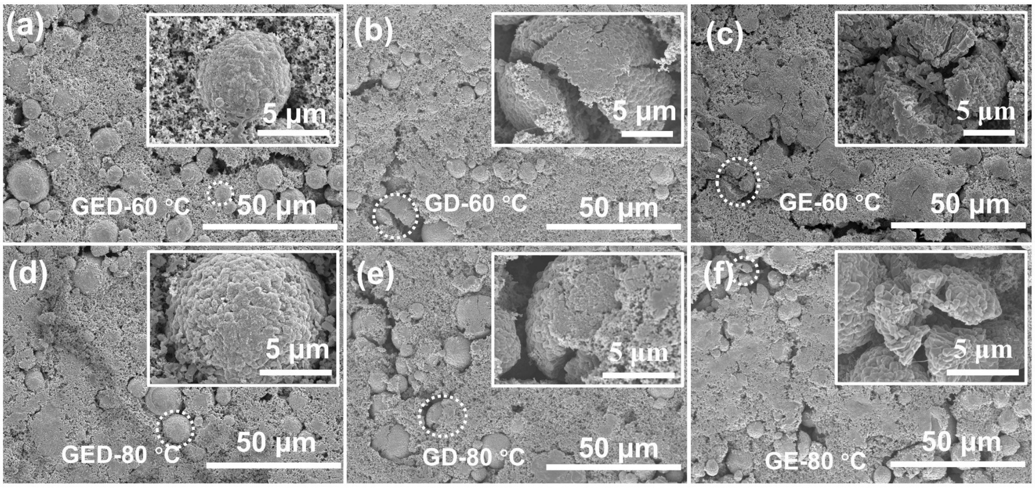

Figure S40. SEM images of cycled NCM811 electrodes with different electrolytes at 60 and 80 °C (a-f).

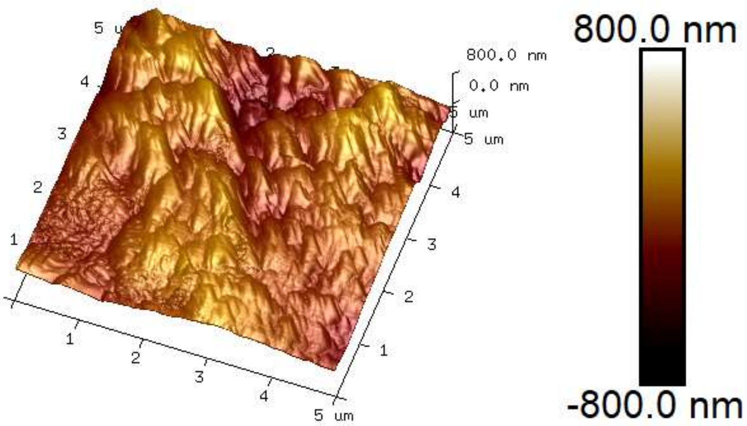

Figure S41. AFM image of pristine NCM811 electrode, Ra = 75.0 nm.

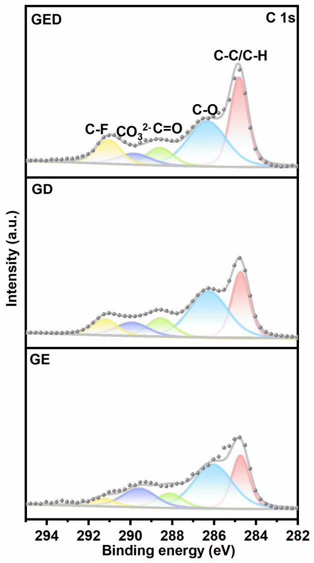

Figure S42. XPS investigation of C 1s of the NCM811 surface with various electrolytes after 30 cycles at 25 °C.

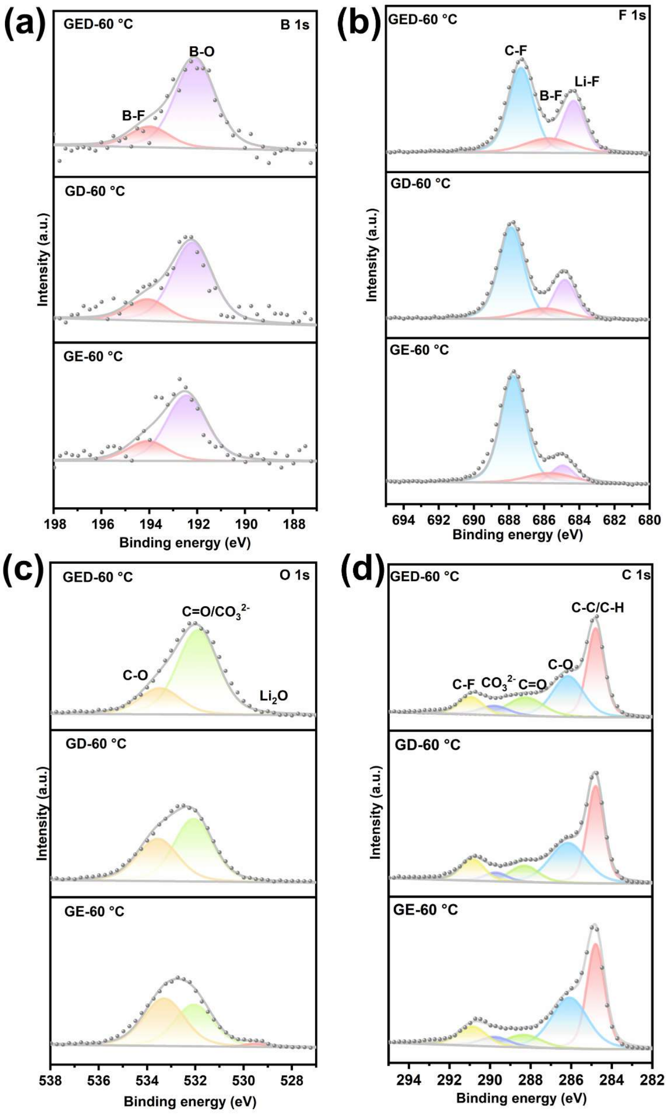

Figure S43. XPS investigation of (a) B 1s; (b) F 1s; (c) O 1s; (d) C 1s of the NCM811 surface with various electrolytes after 30 cycles at 60 ℃.

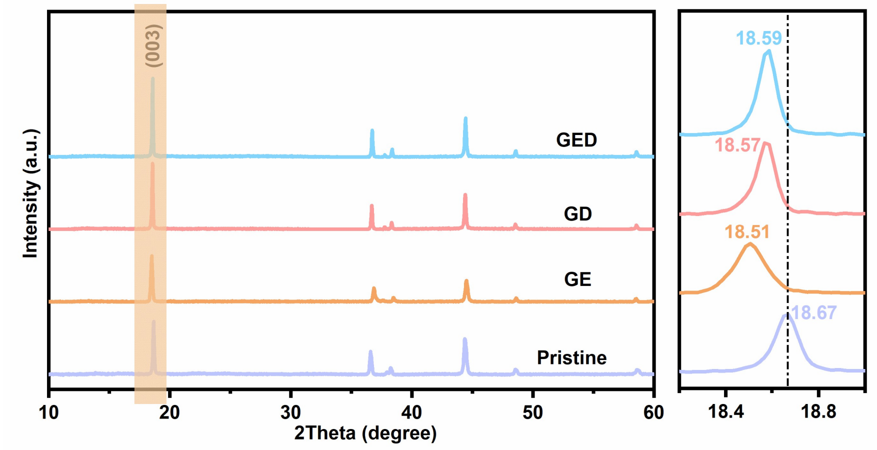

Figure S44. XRD characterization of NCM811 cathodes cycled before and after 200 cycles.

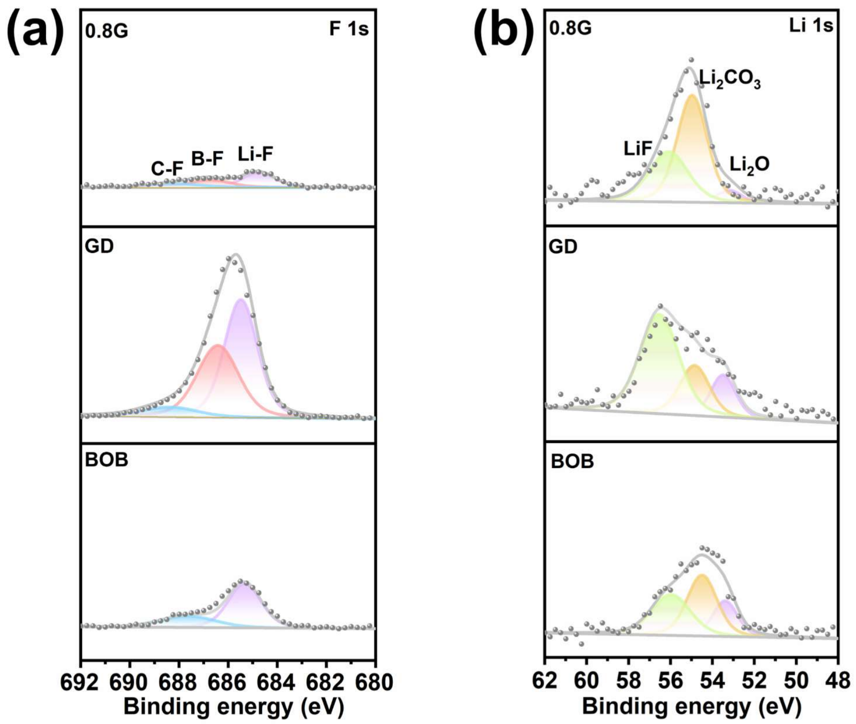

Figure S45. XPS investigation of (a) F 1s and (b) Li 1s of the Li metal surface with various electrolytes after 10 cycles at 25 °C.

Note: As shown in the figure, the characteristic LiF signal of the sample containing lithium difluoro(oxalate)borate (LiBOB) is significantly weaker than that of the GD sample. This directly confirms that LiF in the electrolyte designed in this study is mainly derived from the decomposition of $ODFB^-$ anions. In addition, the characteristic LiF signal of the 0.8 G sample is almost negligible compared with that of the GD sample, which is in high agreement with the molecular dynamics (MD) simulation results described earlier in this paper. In the 0.8 G electrolyte system, due to the absence of DFEC-mediated solvation structure regulation, the degree of $ODFB^-$ anion

participation in the construction of the solvation sheath is extremely low, making it difficult to detect the characteristic LiF peak generated by its decomposition.

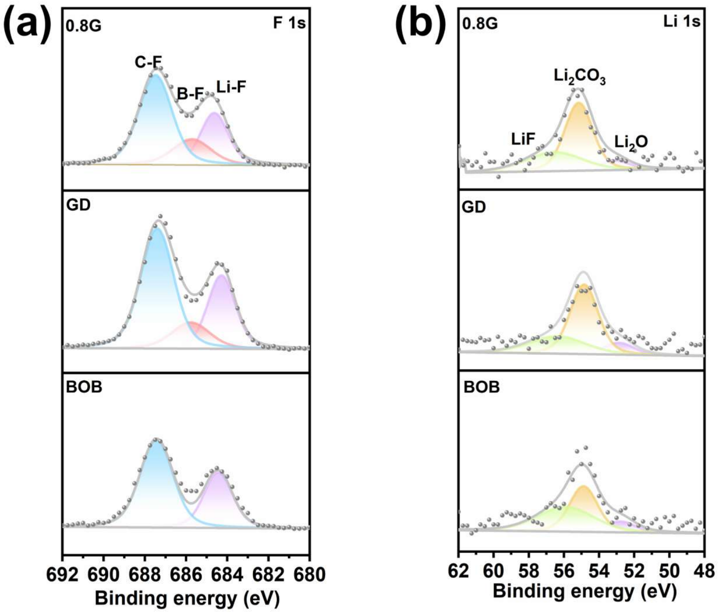

Figure S46. XPS investigation of (a) F 1s and (b) Li 1s of the NCM811 surface with various electrolytes after 10 cycles at 25 ℃.

Table S1. Conductivity and viscosity of different electrolytes (T=25 °C)

Sample	Average Conductivity ($mS \cdot cm^{-1}$)	Viscosity ($mPa \cdot s^{-1}$)
CE	11.56	2.02
0.8G	7.91	3.84
GE	6.13	2.05
GD	6.84	4.21
GED	4.57	2.21

Table S2. Comparison of our work with other recently reported wide-temperature high-voltage LMBs electrolytes.

Ref	Electrolyte	Cathode/anode	Temperature range (°C)	Cut-off voltage (V)	Cycling condition & capacity retention	Year
This work★	0.8M LiODFB in GVL/ETFA/DFEC(3/5/2)	NCM811/Li	-60-100	2.8-4.5	-60 °C, 0.1 C discharge@90.4 mAh·g^{-1}; 100 °C, 90th , 79.4%	2025
Ref 1[5]	2.2M LiFSI in BSF/FEC(3/1)	NCM811/Li	-40-55	2.8-4.3 and 2.8-4.4	-40 °C, 0.1 C discharge， 10th, 100% 55 °C, 250th, 86.5%;	2025
Ref 2[6]	1M LiODFB in DMS/ETFA/FEC(4/4/2)	NCM811/Li	-60-25	2.8-4.3	-60 °C, 0.1 C discharge@29.3 mAh·g^{-1}; -40 °C, 0.1 C, 200th@126.3 mAh·g^{-1}, ~100%	2025
Ref 3[7]	1M $LiPF_6$ in EA/FEC（10/1）+2 vt% PS	NCM811/Li	-40-60	3-4.3	-30 °C, 0.1 C, 200th@95 mAh·g^{-1} 45 °C, 120th, ＜ 80%	2024
Ref 4[8]	LiFSI/TEOS/TTE(molar ratio:1:1.5:1.3)	NCM811/Li	28-60	2.8-4.3	28 °C, 0.5 C, 300th, ~100%; 60 °C, 0.5 C, 200th, 97.5%	2024
Ref 5[9]	0.1M LiODFB in [MEMP][TFSI]/HFE(1:2)	NCM622/Li	-60-70	2.8-4.5	-60 °C ,0.1 C discharge@115 mAh·g^{-1}; 70 °C, 70th, no mentioned	2022
Ref 6[10]	1M $LiPF_6$ in DFEC/DEC	NCM811/Li	-30-20	2.8-4.5	-30 °C， 0.2 C， 50th@93 mAh·g^{-1}， ~100% 20 °C, 300th, 91%	2021
Ref 7[11]	1M LiFSI+0.02M $LiNO_3$ in DME/TTE(3:7)	NCM811/Li	-40-60	2.8-4.3	-40 °C, 0.1C discharge@339 Wh·kg-1 60 °C, 0.1C digcharge@528.0 Wh·kg-1	2025

Ref 8[12]	1.89M LiFSI in DEE/TTE/BTFE(20:20:11)	NCM811/Li	-60-55	3-4.3	-60 ℃, 0.2C discharge@128 $mAh\cdot g^{-1}$; 55 ℃, 100th, 100%	2025
Ref 9[13]	LiFSI in CPME	LFP/Li	-20-25	2.8-3.8	-20 ℃, 0.3 C, 50th, no mentioned 25 ℃, 0.5C, 400th, 90%	2023
Ref 10[14]	LiTFSI/PC/SN(molar ratio of 1:3:3)+0.4wt%$LiNO_3$	NCM523/Li	-10-60	3-4.5	-10 ℃, 0.2C, 100th, 100% 60 ℃,100th, 90.05%	2025
Ref 11[15]	0.8M LiTFSI+0.2M LiODFB+0.01M LiPF6 in EC/PC(1:1)	LCO/Li	25-80	3-4.2	80 ℃, 100th,90%	2019
Ref 12[16]	LiFSI/KFSI/CsFSI(30%:35%:35%)	NCM811/Li	25-80	3-4.3	80 ℃, 280th, 75%	2025
Ref 13[17]	3M LiFSI+0.2M LiODFB in TBP/DME(7:3)	NCM811/Li	25-60	2.8-4.4	60 ℃, 100th, no mentioned	2024

Reference

[1] Y. Zhao, T. Zhou, T. Ashirov, M. E. Kazzi, C. Cancellieri, L. P. H. Jeurgens, J. W. Choi, A. Coskun, *Nat. Commun.* **2022**, *13*, 2575.

[2] Y. Chen, L. Wang, T. Anwar, Y. Zhao, N. Piao, X. He, Q. Zhu, *Electrochim. Acta* **2017**, *241*, 132.

[3] S. Amzil, Y. Xiao, D. Ma, J. Li, T. Xu, Z. Ru, L. Cao, M. Yang, S. Luo, M. Wu, M. Peng, Y. Li, S. Tian, J. Gao, Y. Yu, P. Müller-Buschbaum, T. Cai, F. Zhao, Q. Li, Y.-J. Cheng, Y. Xia, *Materials Science and Engineering: R: Reports* **2025**, *166*, 101051.

[4] X. Zhang, X. Chen, X. Cheng, B. Li, X. Shen, C. Yan, J. Huang, Q. Zhang, *Angew. Chem. Int. Ed.* **2018**, *57*, 5301.

[5] W. Yang, J. Cai, C. Xu, A. Chen, Y. Wang, Y. Shi, P. He, H. Zhou, *Adv. Mater.* **2025**, *37*, 2505285.

[6] H. Zhang, X. Wu, W. Kong, M. Huang, Y. Xue, H. Xiang, Z. Huang, *Energy Storage Mater.* **2025**, *74*, 103955.

[7] Y. Li, B. Wen, N. Li, Y. Zhao, Y. Chen, X. Yin, X. Da, Y. Ouyang, X. Li, P. Kong, S. Ding, K. Xi, G. Gao, *Angew. Chem. Int. Ed.* **2025**, *64*, e202414636.

[8] T. Meng, S. Yang, Y. Peng, P. Li, S. Ren, X. Yun, X. Hu, *Adv. Energy Mater.* **2025**, *15*, 2404009.

[9] Z. Wang, H. Zhang, J. Xu, A. Pan, F. Zhang, L. Wang, R. Han, J. Hu, M. Liu, X. Wu, *Adv. Funct. Mater.* **2022**, *32*, 2112598.

[10] Z. Wang, Z. Sun, Y. Shi, F. Qi, X. Gao, H. Yang, H. Cheng, F. Li, *Adv. Energy Mater.* **2021**, *11*, 2100935.

[11] Z. Xiao, X. Liu, F. Hai, Y. Li, D. Han, X. Gao, Z. Huang, Y. Liu, Z. Li, W. Tang, Y. Wu, S. Passerini, *Angew. Chem. Int. Ed.* **2025**, *64*, e202503693.

[12] X. Zhang, T. Yang, Z. Huang, Q. Zhang, S. Jia, J. Kang, C. He, N. Zhao, Y. Zhang, Z. Chen, *ACS Energy Lett.* **2025**, *10*, 4428.

[13] H. Zhang, Z. Zeng, F. Ma, Q. Wu, X. Wang, S. Cheng, J. Xie, *Angew. Chem. Int. Ed.* **2023**, *62*, e202300771.

[14] W. Zhang, Z. Zhang, H. Zhang, Y. Luo, X. Liu, Z. Rao, *Adv. Sci.* **2025**, *12*, 2416656.

[15] Z. Geng, J. Lu, Q. Li, J. Qiu, Y. Wang, J. Peng, J. Huang, W. Li, X. Yu, H. Li, *Energy Storage Mater.* **2019**, *23*, 646.

[16] S. Yan, N. Yao, H. Liu, Z. Zhang, Y. Lu, Z. Liu, W. Hou, P. Zhou, H. Zhou, X. Chen, K. Liu, Q. Zhang, *Energy Environ. Sci.* **2025**, *18*, 1696.

[17] L. Liao, Z. Han, X. Feng, P. Luo, J. Song, Y. Shen, X. Luo, X. Li, X. Wen, B. Yu, J. Chen, B. Guo, M. Wang, Y. Huang, H. Zhang, M. Yin, J. Liu, Y. Lin, X. Li, *J. Energy Chem.* **2024**, *97*, 156.
